# Use of Sine Shaped High-Frequency Rhythmic Visual Stimuli Patterns for SSVEP Response Analysis and Fatigue Rate Evaluation in Normal Subjects

**DOI:** 10.3389/fnhum.2018.00201

**Published:** 2018-05-28

**Authors:** Ahmadreza Keihani, Zahra Shirzhiyan, Morteza Farahi, Elham Shamsi, Amin Mahnam, Bahador Makkiabadi, Mohsen R. Haidari, Amir H. Jafari

**Affiliations:** ^1^Department of Medical Physics and Biomedical Engineering, School of Medicine, Tehran University of Medical Sciences, Tehran, Iran; ^2^Research Center for Biomedical Technologies and Robotics, Tehran University of Medical Sciences, Tehran, Iran; ^3^Department of Biomedical Engineering, Faculty of Engineering, University of Isfahan, Isfahan, Iran; ^4^Section of Neuroscience, Department of Neurology, Faculty of Medicine, Baqiyatallah University of Medical Sciences, Tehran, Iran

**Keywords:** brain computer interface, SSVEP, EEG, high frequency, rhythmic patterns, sine waves, fatigue rate

## Abstract

**Background:** Recent EEG-SSVEP signal based BCI studies have used high frequency square pulse visual stimuli to reduce subjective fatigue. However, the effect of total harmonic distortion (THD) has not been considered. Compared to CRT and LCD monitors, LED screen displays high-frequency wave with better refresh rate. In this study, we present high frequency sine wave simple and rhythmic patterns with low THD rate by LED to analyze SSVEP responses and evaluate subjective fatigue in normal subjects.

**Materials and Methods:** We used patterns of 3-sequence high-frequency sine waves (25, 30, and 35 Hz) to design our visual stimuli. Nine stimuli patterns, 3 simple (repetition of each of above 3 frequencies e.g., P25-25-25) and 6 rhythmic (all of the frequencies in 6 different sequences e.g., P25-30-35) were chosen. A hardware setup with low THD rate (<0.1%) was designed to present these patterns on LED. Twenty two normal subjects (aged 23–30 (25 ± 2.1) yrs) were enrolled. Visual analog scale (VAS) was used for subjective fatigue evaluation after presentation of each stimulus pattern. PSD, CCA, and LASSO methods were employed to analyze SSVEP responses. The data including SSVEP features and fatigue rate for different visual stimuli patterns were statistically evaluated.

**Results:** All 9 visual stimuli patterns elicited SSVEP responses. Overall, obtained accuracy rates were 88.35% for PSD and > 90% for CCA and LASSO (for TWs > 1 s). High frequency rhythmic patterns group with low THD rate showed higher accuracy rate (99.24%) than simple patterns group (98.48%). Repeated measure ANOVA showed significant difference between rhythmic pattern features (*P* < 0.0005). Overall, there was no significant difference between the VAS of rhythmic [3.85 ± 2.13] compared to the simple patterns group [3.96 ± 2.21], (*P* = 0.63). Rhythmic group had lower within group VAS variation (min = P25-30-35 [2.90 ± 2.45], max = P35-25-30 [4.81 ± 2.65]) as well as least individual pattern VAS (P25-30-35).

**Discussion and Conclusion:** Overall, rhythmic and simple pattern groups had higher and similar accuracy rates. Rhythmic stimuli patterns showed insignificantly lower fatigue rate than simple patterns. We conclude that both rhythmic and simple visual high frequency sine wave stimuli require further research for human subject SSVEP-BCI studies.

## Introduction

A brain computer interface (BCI) interprets human brain activities as a control signal to make direct communication between the brain and external devices (Alonso et al., 2012). Its application includes improving the quality of life in severely disabled individuals. The BCI-based systems utilize cortical signals for communication (Alonso et al., 2012; Diez et al., 2013). BCIs also work with several modalities, such as electroencephalography (EEG) (Hochberg, 2006; Schalk and Leuthardt, 2011), electrocorticogram (ECoG) (Hochberg, 2006), functional magnetic resonance imaging (fMRI) (Sitaram et al., 2008), and functional near-infrared spectroscopy (fNIR or fNIRS) (Naseer and Hong, 2015). Nowadays, non-invasive modalities such as EEG measurement provide a common solution in BCI studies and applications. P300 (Middendorf et al., 2000; Chang et al., 2016), visual evoked potential (VEP) (Wang et al., 2008; Bin et al., 2009a, 2011; Guger et al., 2012; Nezamfar et al., 2016; Zhao et al., 2017), slow cortical potentials (SCP) (Birbaumer et al., 1999) and sensorimotor rhythms (Pfurtscheller et al., 2000) are the common signals used for EEG-based BCIs.

EEG-steady state visual evoked potential (SSVEP) signal is one of the most promising modalities that has several comparative advantages including high signal to noise ratio (SNR) (Ahn et al., 2016), high information transfer rate (ITR) and minimal requirement for training of subject (Middendorf et al., 2000; Wu et al., 2008; Guger et al., 2012). SSVEP signal is a natural response of brain to periodic visual stimulation in range of 1–90 Hz (Herrmann, 2001). It usually includes a sinusoidal waveform with the same fundamental frequency as of the external visual stimulus, harmonics and occasionally subharmonics, and is mostly observed in occipital and parietal lobes (Zhang et al., 2012a).

In the SSVEP-based BCI, an extensive variety of frequencies have been utilized for visual stimulus generation and it is observed that SSVEP has the strongest amplitude when the flickering frequency is about 15 Hz (Pastor et al., 2003). Stimulus frequencies in the medium (12–25 Hz) and low frequency (up to 12 Hz) ranges give better SNR in SSVEP. However, these are associated with subjective fatigue and discomfort and higher risk of photosensitive epileptic seizures (Pastor et al., 2003; Duszyk et al., 2014). On the other hand, high-frequency (25–50 Hz) stimulus range yield lower amplitude in SSVEP (Diez et al., 2011; Ajami et al., 2018); however, they give stable subjective performance with the passage of time making them suitable for SSVEP-based BCI (Won et al., 2015). Several studies have utilized low to medium range frequencies as these have higher SNR than high frequency range and are easy to detect (Friman et al., 2007; Muller et al., 2008; Ortner et al., 2011). Recent studies have detected SSVEP by using visual stimuli at the range of 30–50 Hz (Zhu et al., 2010; F. Zhang et al., 2015). For example, controlling robotic wheelchair and computer mouse at 37–40 Hz frequencies (Diez et al., 2011, 2013) and using 30–39 Hz frequencies for an optimized simple 7-letter speller (Chabuda et al., 2018).

Several studies have developed methods for the detection of SSVEP at high frequencies. For example, spatial filtering approach has been used for the SSVEP detection at 30, 35, 40, and 45 Hz (Molina and Mihajlovic, 2010). In addition, empirical mode decomposition (EMD) method has also been used for the SSVEP recognition at 25, 30, 35 Hz (Liu et al., 2014; Zhang et al., 2015).

The information transfer rate of a BCI system is proportional to the number of commands that are simultaneously available for the user (Yuan et al., 2013). In SSVEP paradigm, a narrow band of frequencies is available so as to have low eye fatigue and acceptable SNR. On the other hand, the refreshing rate of monitors is an important limiting factor for the number of available visual stimuli. Therefore, the number of available targeted frequencies for the modulation is an important problem that remains to be solved for benchmark BCI application such as spellers (Zhang et al., 2012b). Some studies have attempted to overcome this problem by using phase information to code more targets (Lee et al., 2010; Jia et al., 2011) or by embedding more LEDs with a low precise frequency resolution interval between the targeted frequencies (Hwang et al., 2012). Others have proposed the sum of two or more frequencies applied simultaneously to a visual stimulus, mostly with square waveform (Srihari Mukesh et al., 2005; Shyu et al., 2010; Hwang et al., 2013). However, as reported in several studies, the use of square waveform results in appearance of odd harmonics in the SSVEP response that can negatively affect the detection accuracy and increases the ambiguity in choosing the best stimulus frequencies for the target recognition (Srihari Mukesh et al., 2005; Shyu et al., 2010; Zhang et al., 2012b, 2015; Zhao et al., 2017).

In addition, the above mentioned limitations have been addressed by considering specific aspects of challenges. For example, use of high frequency for the reduction of fatigue (Sakurada et al., 2015); however, it decreases the BCI performance (Volosyak et al., 2011). As it is important to reduce fatigue rate and also have high accuracy rate for a real BCI application, several studies have used high frequency for fatigue reduction and low frequencies for improving the accuracy rate by introducing amplitude and frequency modulated visual stimulation methods (Chang et al., 2014; Dreyer and Herrmann, 2015; Dreyer et al., 2017). In these studies, a high frequency carrier was modulated by another frequency and the SSVEP was evoked at the difference of these two frequencies that covered low frequency range for improving the accuracy rate. This method reduced subjective perceptibility as well as fatigue rate; however, the accuracy rate in these studies was lower than the simple stimulus used in recent studies (Dreyer and Herrmann, 2015; Dreyer et al., 2017).

In this study, we used sequence of 3 different frequencies as high frequency rhythmic patterns for the reduction of subjective fatigue rate. We hypothesized that the accuracy of rhythmic patterns would be comparable with the recently used simple visual stimuli. Thus, we designed high frequency simple and rhythmic sine wave patterns emitted by LED with high precision and low total harmonic distortion (THD) rate. We also investigated the accuracy by using three different frequency detection algorithms. Finally, the discrimination of patterns was evaluated by the analysis of SSVEP responses and the subjective fatigue for the simple and rhythmic visual stimuli by using VAS.

## Materials and methods

### Study participants

Twenty-two healthy right handed (11 males and 11 females students; 23–30 years old, average age 25 ± 2.1 years) subjects with normal or corrected to normal vision and without any history of neurologic and psychiatric disorders and head trauma were enrolled in this study. They were recruited by the word of mouth or announcement via social media. All of them signed informed consent form based on the Declaration of Helsinki. The ethical approval for the study was given by the Medical Research Ethics Committee of the Tehran University of Medical Sciences.

### Experimental setup

#### Stimuli design paradigm

In the human visual system, three parallel pathways including Magnocellular (MC), Parvocellular (PC) and Koniocellular (KC), process and transfer visual information to visual cortex (Kaplan, 2014). Each pathway processes different physical parameters of visual stimuli characterized by their specific temporal and spatial resolutions (Purpura et al., 1990; Duszyk et al., 2014; Labecki et al., 2016). Magnocellular pathway is sensitive to difference in contrast and motion and depth information. Receptive fields of MC pathway neurons are larger than other pathways and are sensitive to quick transients in retinal stimulation. In other words, it is sensitive to high-frequency visual stimuli (Cheong et al., 2013; Duszyk et al., 2014). Information about red and green colors is carried by the PC pathway and it is also sensitive to the shape of stimulus and exhibits sustained response to retinal stimulation. Koniocellular pathway carries blue and yellow color information and responds to the spectrum of stimuli (Kaplan, 2014). Based on the above characteristics of human visual system, we expected high SSVEP generation by MC pathway because of its larger receptive fields, its relevant processing of visual stimuli and motion perception. The most common colors used for BCI applications are green, blue, and red and white (Zhu et al., 2010). For choosing the best color to have strong SSVEP response, we selected red color based on PC pathway sensitivity and for obtaining sustained SSVEP response (Zhu et al., 2010; Duszyk et al., 2014).

**Figure 1:**
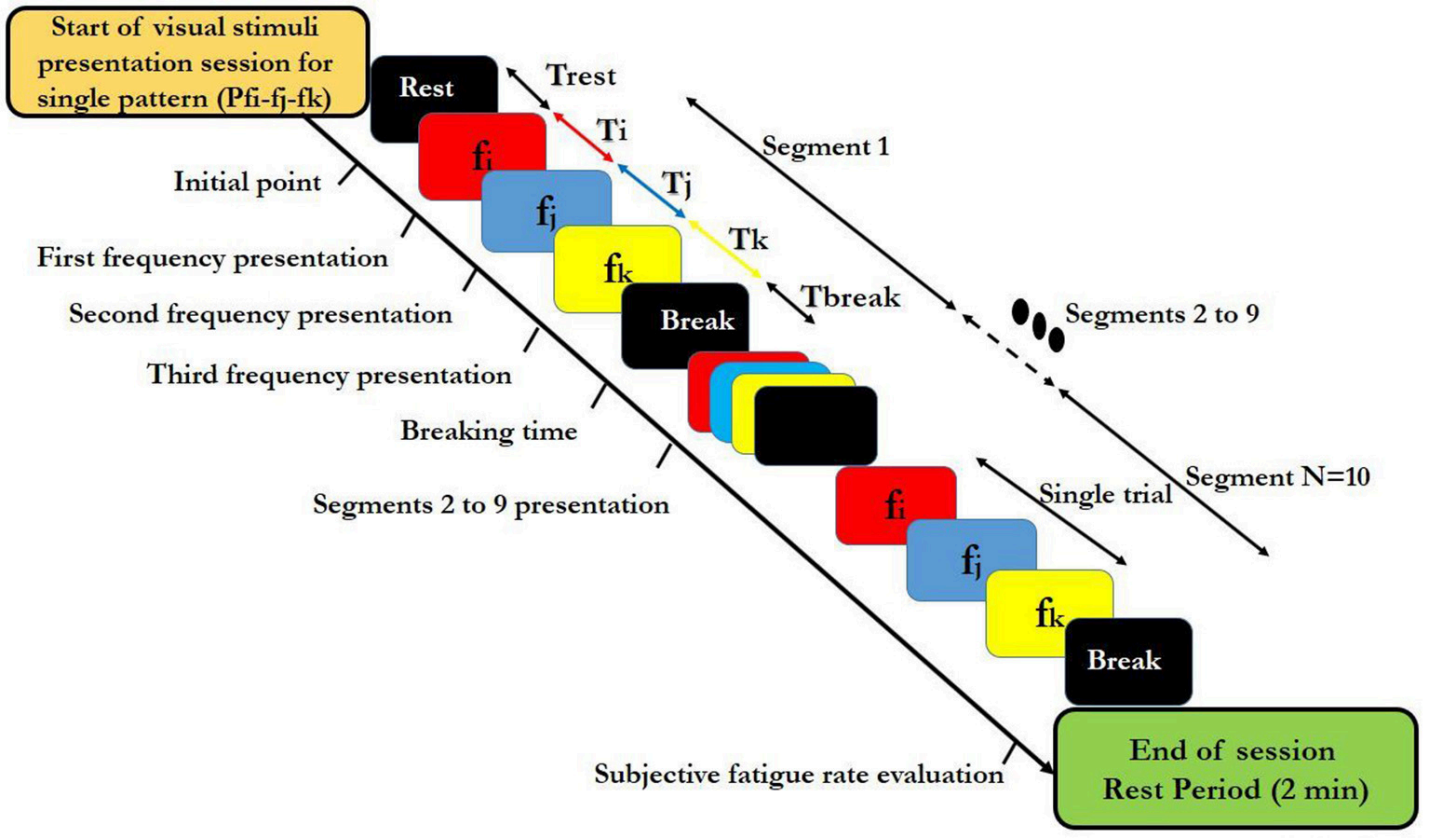
Block diagram of designated high-frequency visual stimuli patterns used in the study. Colors in each segment indicate different frequencies. i,j,k indices relate to individual high frequencies that were used within a segment. Ti, Tj, and Tj show duration of representation of each fi, fj, and fk frequency respectively, in a segment and Trest expresses the rest time before presentaion of each single session. N is the total number of segments in the designed visual stimuli presentation session for a single pattern and shows the repetition number of a single segment. Tbreak denotes the break time between two consecutive segments. A trial contains the sequence of three frequencies fi,fj, and fk presentation. The duration of a visual stimuli presentation session for a single pattern was calculated by following equation: T_session_ = T_rest_ + (T_Trial_ + T_break_) × N.

Knowledge about the shape and size of stimuli presenting device is also crucial for designing a BCI-paradigm. Firstly, we selected LED because of the limitation of the refresh rate of LCD (Zhao et al., 2017). The size of square shaped LED was 4 × 4 cm^2^. Rectangular, square or checkerboard are the most common shapes used in BCI studies and choosing any one of them, have no significant effects on response (Duszyk et al., 2014). Additionally, as increasing the size of LED evokes stronger SSVEP response, we kept 4 × 4 cm^2^ size which was acceptable for use in SSVEP-BCIs (Duszyk et al., 2014). According to our paradigm, each high-frequency visual pattern was coded by the permutation of frequencies that are available in the [25–50 Hz] range. By choosing N frequencies from the frequency set, we could get N^N^ different patterns. These patterns contained N same frequencies, (N^N^-N-N!) partly different and N! completely different frequencies. General block diagram for designing visual stimulus is shown in Figure 1.

**Figure 2:**
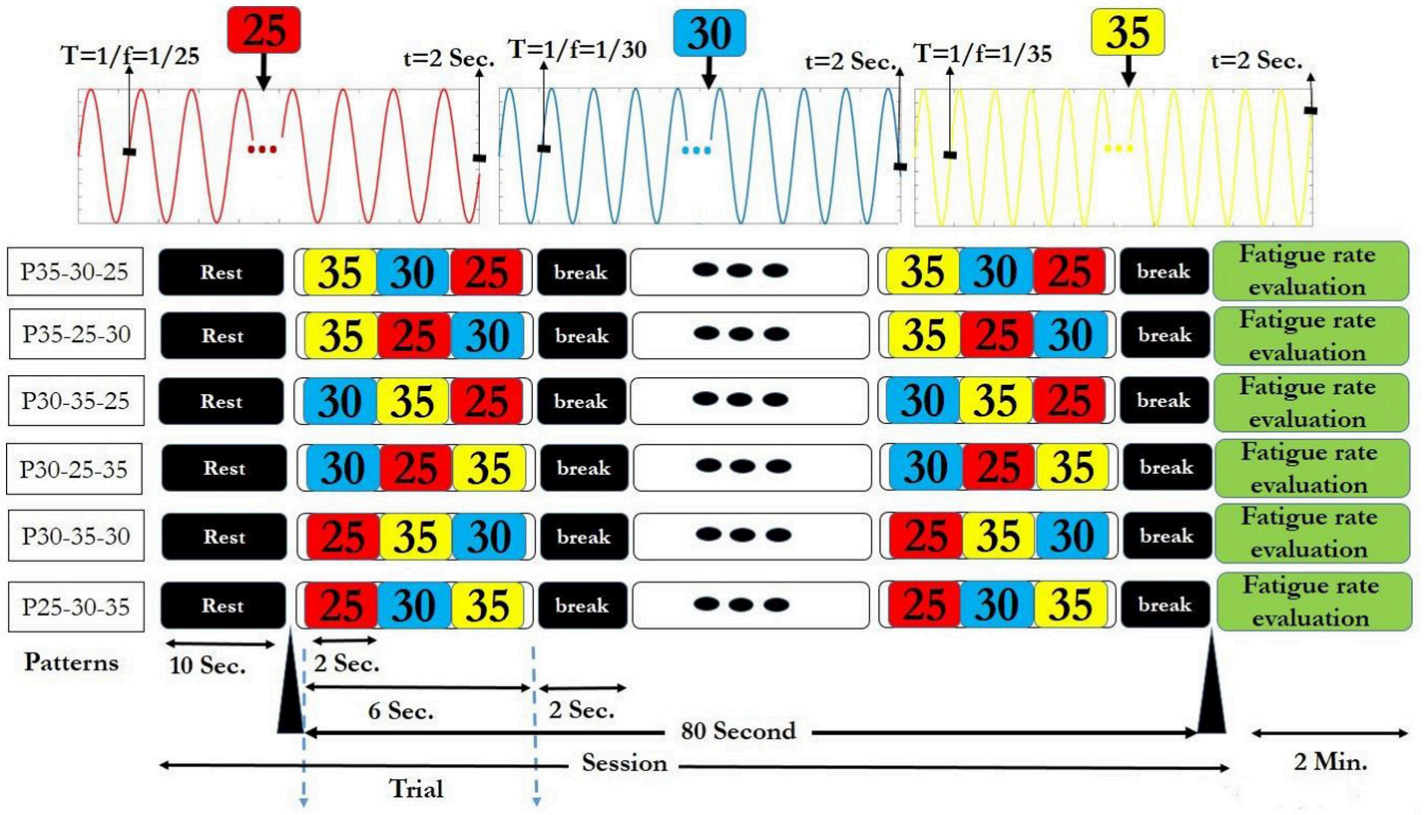
Six rhythmic visual stimuli presentation session. In each session, after a 10 s rest time, the selected pattern was presented as a trial for 6 s. Between the two consecutive trials, a 2 s break time was given. Therefore, each session that contained 10 trials took 90 s. At the end of each session, fatigue rate was reported by the subjects. The session finished after 2 min rest period.

**Figure 3:**
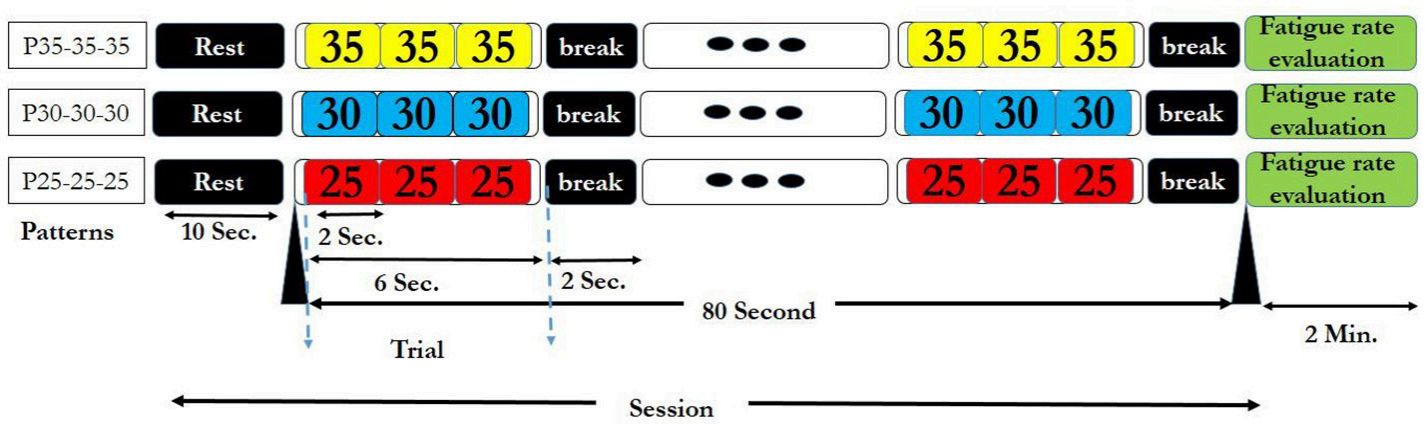
Three simple visual stimuli presentation session. The details of the session are same as of the Figure 2.

For applying the above visual stimuli in our SSVEP study, three high frequencies 25, 30, and 35 Hz were selected to generate various patterns. We generated 9 patterns, (each having 3 frequencies) from 27 possible ones. Three patterns comprising of repetitions of same frequencies (P25-25-25, P30-30-30, and P35-35-35) served as the simple ones (Garcia, 2008). The other six patterns that served as rhythmic patterns, comprised of all of 3 frequencies in different sequences (P25-30-35, P25-35-30, P30-35-25, P30-25-35, P35-30-25, and P35-25-30). Use of the 3 frequencies in the 6 above sequences gave maximum frequency changes and unpredictability in rhythmic patterns. These 6 rhythmic patterns also have ascending (e.g., P25-30-35) descending (e.g., P35-30-25) and zigzag (e.g., P30-25-35) trends in frequencies. The order of presentation of simple and rhythmic visual stimuli pattern was random with 3 simple patterns interspersed between 6 rhythmic patterns and same for all the subjects as shown in Supplementary Table S1. Thus, we were able to investigate the effect of maximum frequency changes on subjective fatigue rate between simple and rhythmic group patterns. Break time of 2 s was placed between the trials for better adaptability. The time series diagram of visual stimuli presentation session for each simple and rhythmic patterns is shown in Figures 2, 3 respectively.

### Stimulator design

**Figure 4:**
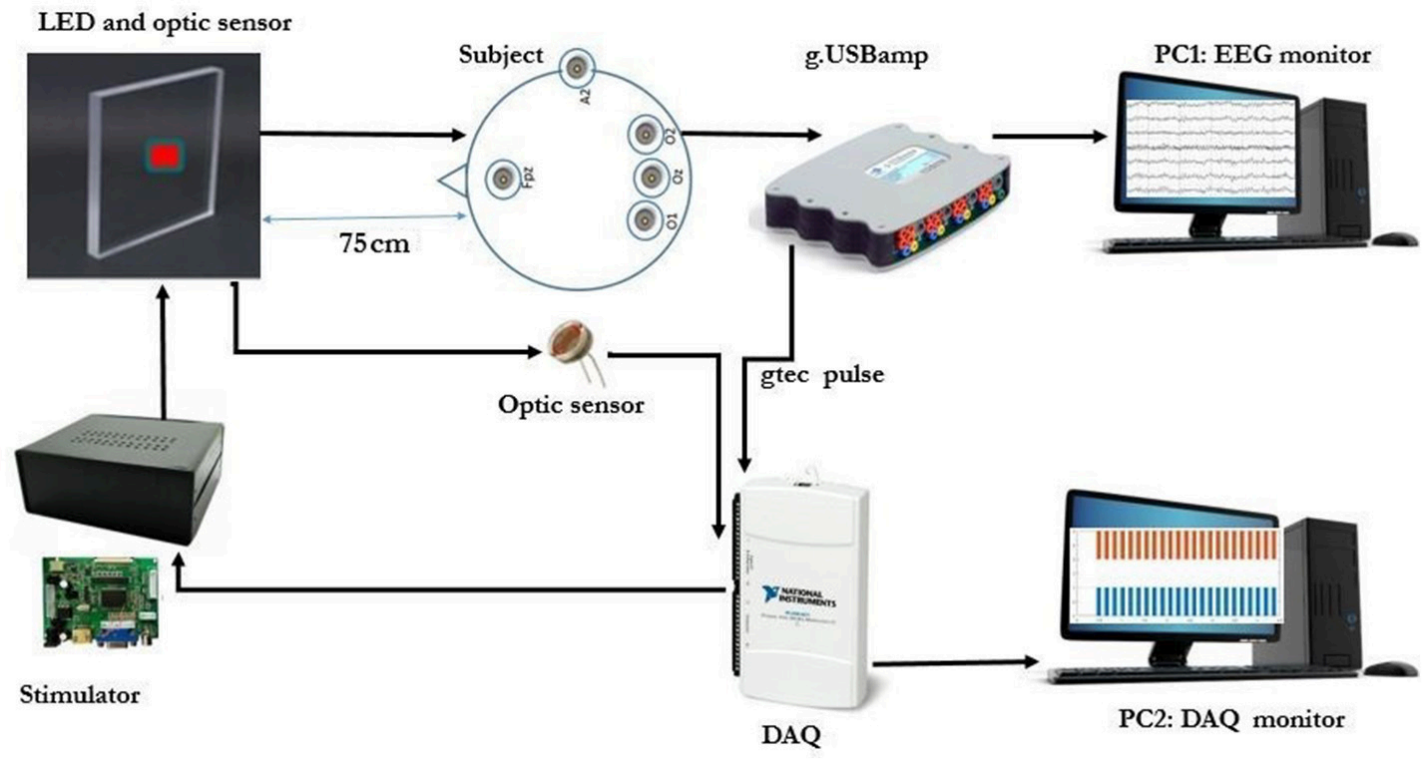
Equipment and flow chart for data acquisition.

**Figure 5:**
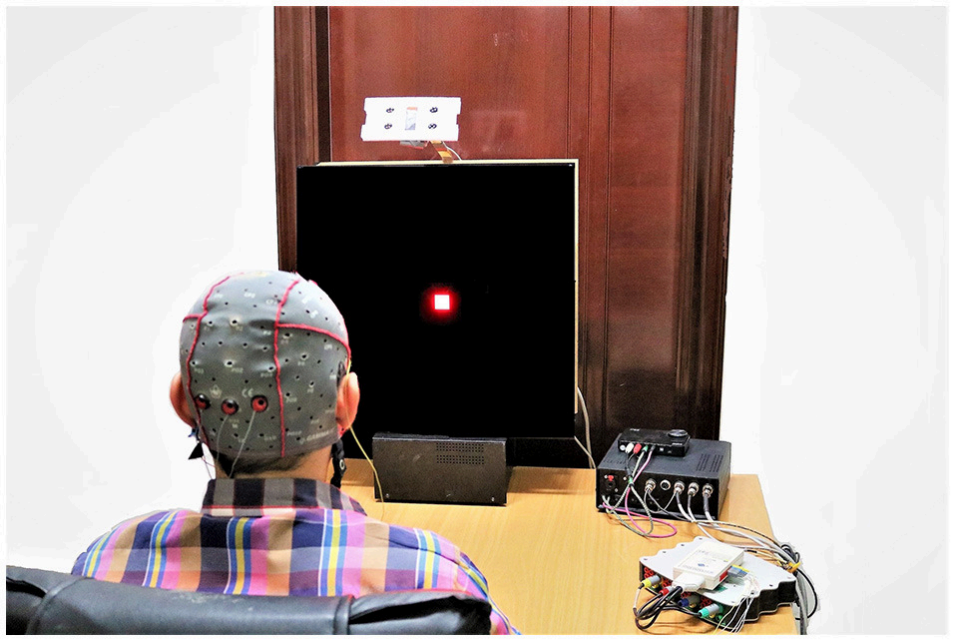
The SSVEP recording experimental setup. (Note: The room light was turned on to capture this image. The SSVEP data were recorded with room light turned off).

All patterns were first designed and entered in MATLAB software (Release 2016b, The MathWorks, Inc, Massachusetts, United States) with the sampling rate of 44100 sps and saved as .wav format to be sent to a custom-made DAC board and LED driver (Figures 4, 5). A precise DC bias was added to the sine waveform pattern with appropriate amplitude to lay in the linear region of operation of the LED. For the LED that was utilized in our setup, the linear region range was 0 to 60 milliamperes with 5.5 Volt DC bias. The stimulator hardware was designed by the authors (MF., AM., BM., and AJ.) so that all the parameters were precisely adjusted, and finally the THD rate of ≤0.1% was achieved (see Supplementary Figures S1, S2). The National Instruments DAQ was used for recording optic sensor signal and the stimuli that were represented concurrently. A linear optical sensor (Texas Instruments) was used for recording the represented stimuli signal from LED (Figures 4, 5).

As shown in the Supplementary Figures S1, S2, the linearity of luminance was analyzed by placing the optical sensor connected to the stimulator hardware (specifications not mentioned due to the pending patent) in front of the LED. We produced a single pure 20 Hz sinusoidal signal with the designed hardware. This signal was applied to the LED with a precision driver. The LED output (e.g., luminosity) was measured with the linear optical sensor. The sensor output was analyzed to calculate the THD of the stimuli.

#### EEG data acquisition and subjective fatigue evaluation

Before starting the experiment, the data acquisition paradigm was explained to each subject. The experiment was carried out in a dimly lit room without electromagnetic shield, and the subjects sat on the comfortable chair at a distance of about 75 cm from the LED screen as shown in the Figures 4, 5. The order of visual stimuli presentation was same for all the subjects (see Supplementary Table S1). They were given 2 minrest after each session. Subjective fatigue rate was evaluated during the rest time by using a 10 point (0 = no fatigue and 10 = maximum fatigue) Visual Analogue Scale (VAS) (Shahid et al., 2011). The EEG signals were recorded continuously by a g.USB amplifier (g.tec, Graz, Austria) system using g.LADYbird active electrodes at Oz, O1, and O2 connected to 3 channels (Ch1, Ch2 and Ch3) for the reported strong SSVEP response. Right ear lobe and Fpz electrodes were selected as reference and ground respectively, by following the EEG international 10–20 standard system and all the 3 channels were sampled at 1200 Hz sampling rate (Figures 4, 5).

### Analytical methods

#### Pre-processing

Based on the specifications of the optic sensor and synchronizer gtec.'s pulse signals, the initial time point of EEG recording was marked by gtec.'s synchronized pulse and the onset time of the stimuli presentation was determined by the recorded optic sensor signal. Finally, the beginning time point of EEG signal with respect to the visual stimuli representation was calculated by the subtraction of optic sensor initial point from gtec.'s pulse onset. Therefore, each segment of EEG-SSVEP was separated. We checked the signals visually and removed trend of data with detrend command in the MATLAB software. Single trials and mean of 10 trials were calculated for each pattern in order to utilize them for further respective single and mean trials data analysis.

#### PSD based analysis

SSVEP signals were filtered by offline 6-order Butterworth band-pass filter with cut-off frequencies of 10–40 Hz, (Diez et al., 2011). The power spectral density was calculated at each 2 s rectangular window, then normalized at each visual stimulus frequencies (25, 30, 35, and Hz) as:

(1) $$P\left(f_{i}\right)=\sum_{ch=1}^{M}\frac{PSDssvep\left(f_{i}\pm 0.25\;Hz\right)}{PSDrest\left(f_{i}\pm 0.25\;Hz\right)}/M$$

Where *ch* is the number of channels, *M* is the total number of channels, *f*_*i*_ is the visual stimuli frequencies, *PSDssvep* is the power spectral density of EEG-SSVEP signal and *PSDrest* is the power spectral density of resting state EEG signal. Finally, *max P* (*f*_*i*_) utilized for recognition of target stimuli as:

(2) $$f_{target}\;=\;max_{f_{i}}\left(\;P_{1}.\;P_{2}.\ldots .\;P_{i}\right)$$

#### Canonical correlation analysis (CCA)

CCA basically works on two random sets of data that may have underlying correlation. The validity, and effectiveness of CCA has been shown in Bin et al. (2009b) study. In our study, first set of data was EEG-SSVEP signal and the second data set was visual stimulus signals. The assumption for utilizing CCA feature extraction method for SSVEP detection is that the source of SSVEP signal (x) is the output of a linear system with visual stimulus (Y) as the input signal. Y can be decomposed in Fourier series of its harmonics as:

(3) $$y=\left[\begin{array}{c} sin\left(2\pi ft\right)\\ cos\left(2\pi ft\right)\\ sin\left(4\pi ft\right)\\ cos\left(4\pi ft\right)\\ \sin\left(6\pi ft\right)\\ cos\left(6\pi ft\right) \end{array}\right]\;t=\frac{1}{S}.\frac{2}{S}\;.\ldots .\;\frac{T}{S}$$

Where *f* is the fundamental frequency of visual stimulus, *S* is the sampling rate and *T* is the number of sample points. CCA algorithm finds a pair linear combination for x and y as, *X* = ax and *Y* = by to maximize the correlation between two canonical variables {*X, Y*}, based on the following optimization problem:

(4) maxρ(X.Y)=Corr(X. Y)

(5) $$max\rho \left(X.Y\right)=\frac{E\left[X^{T}Y\right]}{\sqrt{E\left[X^{T\;}X\right]E\left[Y^{T\;}Y\right]}}$$

The ρ_*i*_ coefficients utilized as the canonical correlation coefficients for detection of targeted visual stimulus frequency as:

(6) $$f_{target}=max_{f_{i}}\left(\rho_{1}.\;\rho_{2}.\ldots .\;\rho_{i}\right)$$

For more details about the CCA we referred to Lin et al. (2007).

#### Least absolute shrinkage and selection operator analysis (LASSO)

LASSO as a method for recognition of SSVEP was proposed by Zhang et al. (2012a). This analysis has the capability of model selection and shrinkage estimation methodology. Due to its sparse approximation constraints, this method provides low variance and high interpretable solution for linear regression. By considering a standard linear regression model that y represents in the SSVEP response observations as:

(7) y=Xβ+ε

Where the size of *y* is n×1 vector, *X* is a n×p matrix that demonstrates stimuli frequencies and their harmonics and ε denote noise vector with zero mean and constant variance. The LASSO solves the below optimization problem for estimating sparse $\tilde{\beta }$ vector as:

(8) $$\tilde{\beta }=arg\;min(||y-X\beta |{|}_{2}^{2}\;+\;\lambda ||\beta |{|}_{1})$$

Where || .||_1_ and || .||_2_ represent *l*_1_ norm and *l*_2_ norm and λ is a penalty parameter that helps to achieve sparse solution for $\tilde{\beta }$. The contribution degree (*CD*) of each stimulus frequency and its harmonics in EEG response was calculated as:

(9) $$CD_{i}=\;\frac{\sum_{ch=1}^{M}\sum_{h=1}^{2K}\left|{\tilde{\beta }}_{ch.h}^{K}\right|}{M}$$

Where *M* is the total number of EEG channels and K is the number of considered harmonics and ${\tilde{\beta }}_{ch.\;h}^{ch}$ is the $\tilde{\beta }$ = [${\tilde{\beta }}_{1.1}^{1}$, ${\tilde{\beta }}_{1.2}^{1}$, ${\tilde{\beta }}_{1.3}^{1}$, ${\tilde{\beta }}_{1.4}^{1}$, ${\tilde{\beta }}_{1.5}^{1}$, ${\tilde{\beta }}_{1.6}^{1}$,…, ${\tilde{\beta }}_{3.5}^{3}$, ${\tilde{\beta }}_{3.6}^{3}$] respect to each channel and harmonics. Finally, the maximum *CD*_*i*_ is selected as targeted stimuli as:

(10) $$f_{target}=max_{f_{i}}\left(CD_{1}.\;CD_{2}.\ldots .\;CD_{i}\right)$$

### Evaluation methods

Classification accuracy was utilized for evaluating the discriminability of the patterns proposed in this study for SSVEP-BCI applications. For the mean of 10 trials classification, mean of 10 trials of SSVEP response of each pattern was calculated for each subject. Therefore, the total number of trials (for all nine patterns) was equal to 198 (= 9 patterns × 22 subjects). For single trial classification, the total number of trials was equal to 1980 (= 10 trials for each pattern × 9 patterns × 22 subjects).

Classification accuracy was formulated as:

(11) $$Acc=\frac{Number\;of\;valid\;trials\;}{Total\;number\;of\;trials}\times 100$$

Where valid trials are the trials that were correctly classified.

**Figure 6:**
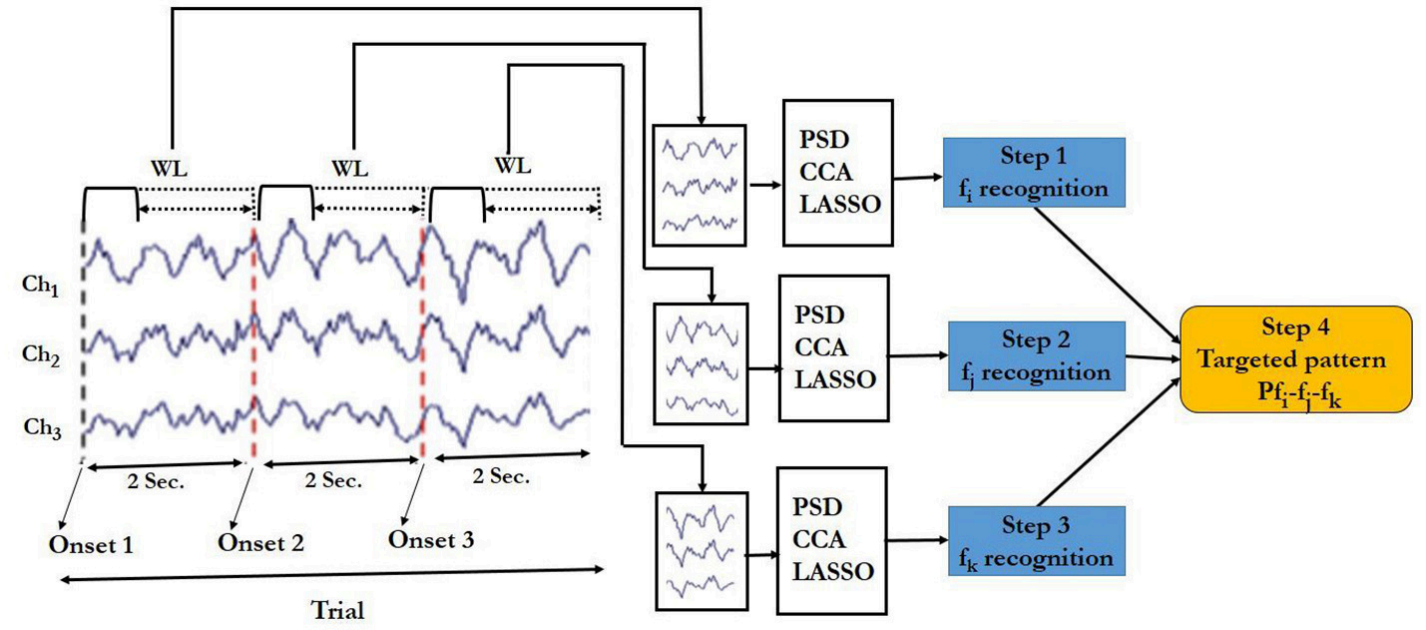
Block diagram of the targeted pattern recognition for SSVEP responses. Ch1 = Oz, Ch2 = O1, Ch3 = O2 are EEG data channels and WL denotes the window length. Dotted line under WL indicates variable window lengths between 0.5 and 2 s.

Classification steps carried out according to the block diagram shown in Figures 6 (see also Supplementary Figure S3) are given below:

Step 1: Target frequency in window 1. The EEG data in window 1 was input of the PSD, CCA or LASSO algorithm to determine the dominated frequency (fi) in the current window.

Step 2: Target frequency in window 2. The EEG data in window 2 was input of the frequency recognition algorithm to determine the dominated frequency (fj) in the current window.

Step 3: Target frequency window3. Similar to that of Step 1 and 2, the dominated frequency (fk) was estimated based on the EEG in window 3.

Step 4: Target pattern recognition. After getting fi, fj and fk, we had the ordered sequence fi-fj –fk. We then compared it with the respective pattern. If it matched with the predefined pattern, the output was taken as 1 or else it was taken as 0.

### Statistical analysis

Statistical analysis was performed with SPSS (Version 16.0. Chicago, SPSS Inc. IBM Corp, released 2011) to evaluate the fatigue rate of 9 patterns used in this study. Appropriate non-parametric statistical tests were employed when fatigue rate data set failed to provide criteria for normal distribution. Therefore, first the Friedman test with a significance level α = 0.05 was employed as non-parametric test to overall demonstrate any significant fatigue rate changes in all visual stimuli patterns as multiple conditions, then the *post hoc* analysis was done to compare and specifically show each pair of patterns fatigue rate changes. Wilcoxon signed-rank test was applied for pairwise comparisons. Bonferroni correction was used because several pairwise comparisons were conducted in the experiment to get the critical value, so that the test had *P* < 0.0014 to be significant. Paired *t*-test was used for comparison of the simple and rhythmic pattern groups fatigue rate (α = 0.05). ANOVA with repeated measures was used to compare the amplitude of CCA and LASSO coefficients of SSVEP responses (α = 0.05) for single trial analysis. Unless mentioned otherwise, the results are expressed as mean ± standard deviation (S.D.). We compared three targeted frequencies coefficient's amplitude and then examined the patterns amplitude changes for simple and rhythmic groups. *Post hoc* analysis has done by using Bonferroni correction (set α < 0.017) in order to check the specific significant differences between each pairs. The CCA and LASSO accuracy results for individual window lengths were analyzed by Wilcoxon signed-rank test with α = 0.05. We statistically compared simple and rhythmic group accuracy results of mean of 10 trials with Wilcoxon signed-rank test (α = 0.05) and single trial accuracy results were compared with paired *t*-test (α = 0.05).

## Results

### Results for mean of 10 trials analysis

**Figure 7:**
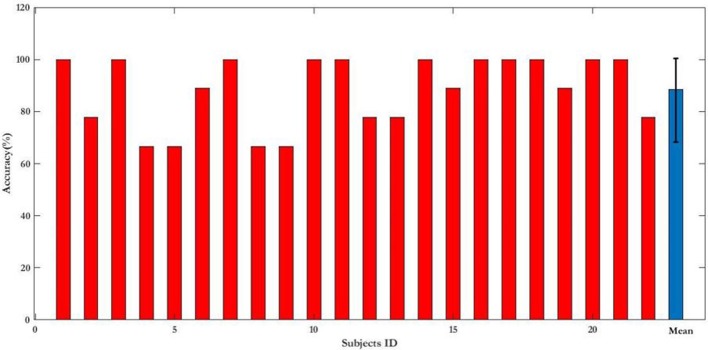
Recognition accuracy expressed as percentage of 22 subjects in PSD-based analysis for 2 s rectangular window length. For single subjects the results are presented as mean. Results of all the subjects (blue bar) is presented as mean ± S.D.

**Figure 8:**
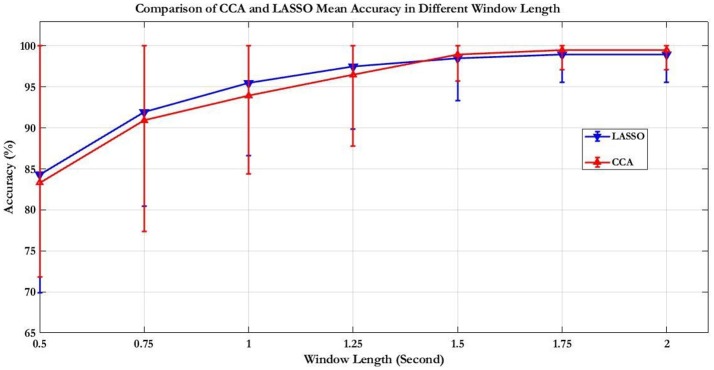
Comparison of CCA and LASSO accuracy results for mean of 10 trials. The results expressed as percentage are presented as mean ± S.D. Comparison of the simple and rhythmic patterns accuracy results of mean of 10 trials showed no statistically significant differences.

First, the PSD-based analysis was performed at an average of 10 trials in 2 s rectangular window and the recognition accuracies for 22 subjects were calculated as shown in Figure 7. For the Grand Average of SSVEP responses and PSD for all nine patterns, see Supplementary Figure S4. Then, the CCA and LASSO analysis were done for 0.5 s window lengths. By increasing the length of window by 0.25 s, the analysis (CCA, LASSO) was repeated for mean of 10 trials at 0.75, 1, 1.25, 1.5, 1.75, and 2 s window lengths as shown in Tables 1, 2 and Figure 8. There was no statistically significant difference between the mean accuracy results of CCA and LASSO.

**Table 1:** CCA accuracy results for different window length (for mean of 10 trials).

**Subjects ID**	***WL* = 0.5 s**	***WL* = 0.75**	***WL* = 1**	***WL* = 1.25**	***WL* = 1.5**	***WL* = 1.75**	***WL* = 2**
S1	66.67	66.67	77.78	88.89	100	100	100
S2	77.78	88.89	66.67	66.67	88.89	88.89	88.89
S3	100	100	100	100	100	100	100
S4	88.89	100	88.89	100	100	100	100
S5	88.89	100	100	100	100	100	100
S6	88.89	100	100	100	100	100	100
S7	88.89	100	100	100	100	100	100
S8	88.89	100	100	100	100	100	100
S9	100	100	100	100	100	100	100
S10	88.89	100	100	100	100	100	100
S11	66.67	100	100	100	100	100	100
S12	55.56	55.56	77.78	77.78	88.89	100	100
S13	88.89	100	100	100	100	100	100
S14	100	100	100	100	100	100	100
S15	77.78	66.67	88.89	100	100	100	100
S16	77.78	88.89	100	100	100	100	100
S17	88.89	100	88.89	100	100	100	100
S18	66.67	88.89	88.89	100	100	100	100
S19	88.89	77.78	100	100	100	100	100
S20	88.89	77.78	100	100	100	100	100
S21	77.78	88.89	88.89	88.89	100	100	100
S22	77.78	100	100	100	100	100	100
Mean(SD)	83.33(11.75)	90.91(13.55)	93.94(9.53)	96.46(8.66)	98.90(3.26)	99.49(2.36)	99.49(2.36)

**Table 2:** LASSO accuracy results for different window length (for mean of 10 trials).

**Subjects ID**	***WL* = 0.5 s**	***WL* = 0.75**	***WL* = 1**	***WL* = 1.25**	***WL* = 1.5**	***WL* = 1.75**	***WL* = 2**
S1	77.78	66.67	77.78	100	100	100	100
S2	88.89	88.89	88.89	88.89	88.89	88.89	88.89
S3	100	100	100	100	100	100	100
S4	88.89	88.89	88.89	88.89	100	100	100
S5	88.89	100	100	100	100	100	100
S6	88.89	100	100	100	100	100	100
S7	77.78	100	100	100	100	100	100
S8	100	88.89	100	100	100	100	100
S9	100	100	100	100	100	100	100
S10	100	100	100	100	100	100	100
S11	77.78	100	100	100	100	100	100
S12	55.56	66.67	66.67	66.67	77.78	88.89	88.89
S13	100	100	100	100	100	100	100
S14	100	100	100	100	100	100	100
S15	77.78	66.67	88.89	100	100	100	100
S16	77.78	88.89	100	100	100	100	100
S17	100	100	100	100	100	100	100
S18	77.78	88.89	100	100	100	100	100
S19	55.56	88.89	88.89	100	100	100	100
S20	88.89	100	100	100	100	100	100
S21	66.67	100	100	100	100	100	100
S22	66.67	88.89	100	100	100	100	100
Mean(SD)	84.34(14.40)	91.92(11.46)	95.45(8.84)	97.47(7.61)	98.48(5.19)	98.99(3.26)	98.99(3.26)

### Single trial analysis results

All the three methods were also used for single trial analysis. The PSD results for single trial WL of 2 s are presented in Table 3. CCA and LASSO analysis results are shown in the Tables 4, 5 for single trial in different WL durations (0.5 to 2 s).

**Table 3:** Single trial analysis accuracy results for PSD method at window length = 2 s.

**Subjects ID**	**Accuracy (%)** ***WL* = 2 s**
S1	36.66
S2	38.14
S3	34.81
S4	40
S5	35.92
S6	35.55
S7	34.07
S8	36.29
S9	35.55
S10	37.40
S11	37.45
S12	32.59
S13	40.37
S14	34.07
S15	36.29
S16	35.92
S17	39.62
S18	33.70
S19	33.70
S20	37.03
S21	32.22
S22	37.03
Mean(SD)	36.06(2.23)

**Table 4:** CCA accuracy results for different window length (single trial).

**Subjects ID**	***WL* = 0.5 s**	***WL* = 0.75**	***WL* = 1**	***WL* = 1.25**	***WL* = 1.5**	***WL* = 1.75**	***WL* = 2**
S1	37.41	41.11	40.00	38.52	36.30	37.78	38.89
S2	46.67	52.96	58.52	65.19	65.93	65.56	67.41
S3	58.52	74.44	82.96	86.67	91.11	93.33	95.56
S4	67.04	77.41	81.85	85.93	90.74	92.59	95.19
S5	40.74	44.81	52.22	56.30	59.26	62.59	65.19
S6	53.70	64.44	70.00	76.67	82.22	85.93	86.67
S7	48.52	65.93	67.41	70.37	74.44	78.89	79.63
S8	36.67	41.11	47.41	55.56	62.22	67.78	70.74
S9	45.93	68.89	77.41	83.70	87.04	90.74	92.59
S10	42.96	65.56	74.07	81.11	82.59	85.56	85.93
S11	49.63	66.67	77.41	83.70	86.30	87.41	88.52
S12	34.44	39.26	42.59	41.85	47.04	47.41	48.89
S13	68.52	78.52	86.30	88.52	92.59	94.07	94.44
S14	53.70	64.81	71.48	74.81	76.30	76.30	80.74
S15	42.22	41.85	46.67	46.67	46.67	47.41	51.11
S16	44.07	50.37	58.52	58.89	58.52	62.59	63.70
S17	48.89	61.11	64.44	72.22	76.67	78.15	81.48
S18	40.74	45.93	53.70	54.07	57.04	57.04	61.11
S19	39.63	43.70	49.26	51.48	52.22	54.44	55.56
S20	45.19	56.67	65.19	74.44	78.89	78.52	85.19
S21	42.96	51.48	57.41	60.37	65.19	67.78	68.52
S22	52.22	61.85	68.52	77.04	77.78	82.96	84.44
Mean(SD)	47.29(8.95)	57.22(12.56)	63.33(13.67)	67.46(15.32)	70.32(16.22)	72.49(16.56)	74.61(16.49)

**Table 5:** LASSO accuracy results for different window length (single trial).

**Subjects ID**	***WL* = 0.5 s**	***WL* = 0.75**	***WL* = 1**	***WL* = 1.25**	***WL* = 1.5**	***WL* = 1.75**	***WL* = 2**
S1	41.41	48.07	50.41	48.89	50.74	52.96	85.56
S2	49.19	56.22	63.63	61.11	64.44	64.81	88.52
S3	58.07	73.63	80.00	80.00	86.67	92.22	45.56
S4	69.44	80.41	88.81	89.63	92.59	94.81	95.56
S5	40.67	50.67	53.11	54.07	58.15	59.26	78.89
S6	58.81	68.44	76.22	77.04	81.85	85.56	51.48
S7	49.56	64.37	68.81	71.11	73.33	75.56	64.44
S8	34.85	41.41	50.67	53.33	61.11	65.19	81.85
S9	50.30	65.85	75.26	80.00	84.44	87.78	58.89
S10	48.81	66.59	76.59	78.15	82.22	84.07	52.22
S11	56.59	62.96	79.19	80.74	84.81	86.67	55.19
S12	39.19	44.74	40.15	40.00	44.07	41.85	81.11
S13	71.41	83.26	87.67	88.89	91.85	92.22	63.70
S14	56.59	66.22	70.15	71.11	74.07	75.56	75.19
S15	45.64	50.67	46.70	49.63	50.37	50.00	66.67
S16	44.74	48.81	55.81	54.81	61.11	60.74	94.07
S17	52.52	62.15	66.96	70.37	77.04	81.48	96.67
S18	44.37	49.56	55.85	52.96	55.56	54.07	63.70
S19	43.26	47.33	48.56	46.30	52.96	53.70	86.30
S20	46.22	55.11	65.48	68.15	72.96	75.56	77.04
S21	44.74	55.48	58.19	58.89	63.33	62.59	67.78
S22	52.63	59.44	61.41	63.33	67.04	70.74	88.89
Mean(SD)	49.95(9.1)	59.15(11.33)	64.52(13.67)	65.39(14.36)	69.58(14.43)	71.25(15.63)	73.60(15.44)

Comparison of the simple and the rhythmic groups visual stimuli was done for single trial and the mean of 10 trials in CCA and LASSO analysis and the respective results are shown in the Table 6. For single trials results, higher accuracy rate was seen for the rhythmic patterns (Table 6 and Supplementary Table S5). Paired *t*-test showed that the single trial accuracy analysis between the simple and the rhythmic groups had statistically significant differences for 2 s WL in both CCA [*t*_(21)_ = −5.689, *P* < 0.001] and LASSO [*t*_(21)_ = −5.086, *P* < 0.001] as shown in Table 6 and Supplementary Table S5.

**Table 6:** Rhythmic and Simple group results comparison for CCA and LASSO.

**Method**	**CCA**		**LASSO**	
**WL (s)**	**2 s**		**2 s**	
**Simple group accuracy (%)**	**Single trial**	**Mean of 10 trials**	**Single trial**	**Mean of 10 trials**
	71.34(17.09)	98.48(7.10)	70.01(16.37)	98.48(7.10)
**Rhythmic group accuracy (%)**	76.24(16.34)	100(0)	75.40(15.23)	99.24(3.55)
**Statistical analysis for comparing the rhythmic and simple group accuracies**	Paired *t*-test (α = 0.05)	Wilcoxon Signed Ranks Test (α = 0.05)	Paired *t*-test (α = 0.05)	Wilcoxon Signed Ranks Test (α = 0.05)
	*t*_(21)_ = −5.689, *P* < 0.001	*Z* = −1.00, *P* = 0.317	*t*_(21)_ = −5.086, *P* < 0.001	*Z* = −0.447, *P* = 0.655

Repeated measures ANOVA with a Greenhouse-Geisser correction showed that the amplitude of coefficients differed statistically significant between the frequency targets in rhythmic group (CCA method: [Target = 25 Hz, *F*_(1.210, 1595.981)_ = 1721.225, *P* < 0.0005], [Target = 30 Hz, *F*_(1.425, 1879.602)_ = 953.149, *P* < 0.0005], [Target = 35 Hz, *F*_(1.648, 2173.479)_ = 340.966, *P* < 0.0005]). (LASSO method: [Target = 25 Hz, *F*_(1.444, 1032.744)_ = 1032.744, *P* < 0.0005], [Target = 30 Hz, *F*_(1.549, 2043.336)_ = 1142.862, *P* < 0.01], [Target = 35 Hz, *F*_(1.863, 2456.753)_ = 425.449, *P* < 0.0005]). *Post hoc* tests using the Bonferroni correction revealed that the targeted 25 Hz frequency was significantly different from 30 to 35 Hz frequencies coefficients ([mean ± *SD* = 0.186 ± 0.083], *P* < 0.001). Multiple comparison results gave repeated similar outputs with the targeted 30 Hz ([mean ± SD = 0.150 ± 0.071], *P* < 0.001) and 35 Hz ([mean ± *SD* = 0.121 ± 0.055], *P* < 0.001) frequencies. These significant results showed that the SSVEP responses to each stimulus frequency were evoked (also see Supplementary Figure S4 that showed the SSVEP responses). We have shown the matrix of mean values of coefficients amplitude (Supplementary Tables S2, S3) that also illustrate differences in the ascending, descending and zigzag trends in rhythmic and the simple patterns. Example of single subject recognized patterns according to CCA coefficients is shown in Supplementary Figure S3.

### Fatigue rate statistical results

Friedman test showed that there was statistically significant difference between fatigue rate of 9 visual stimuli patterns $\chi_{\left(8\right)}^{2}$ = 49.275, *P* = 0.001. *Post hoc* analysis with Wilcoxon signed-rank tests was conducted with application of Bonferroni correction resulting in significance level of *P* < 0.0014. Descriptive statistics is shown in Table 7 and *post hoc* analysis results are shown in Table 8. Overall, there was no significant difference between the fatigue rate of rhythmic patterns group [3.85 ± 2.13] compared to the simple patterns group [3.96 ± 2.21], (*P* = 0.63). However, within the simple patterns group, there was significant variation (min = P35-35-35 [2.95 ± 2.45], max = P25-25-25[4.95 ± 2.57]) of fatigue rate. In addition, within group significant variation of fatigue rate in rhythmic group was observed (min = P25-30-35 [2.90 ± 2.45], max = P35-25-30 [4.81 ± 2.65]) (see Tables 7, 8 and Supplementary Figure S6). For the individual patterns, the maximum fatigue rate (4.95 ± 2.57) and minimum (2.90 ± 2.27) fatigue rate corresponded to P25-25-25 and P25-30-35 patterns respectively. Fatigue rate between the first (P25-30-35) and last (P30-25-35) pattern was not statistically significant (Table 8 and Supplementary Figure S6). However, there was a statistically insignificant trend toward higher fatigue rates at the later time points (Supplementary Figures S6 and S7).

**Table 7:** Descriptive statistics of two groups visual stimuli patterns fatigue rate.

**Groups**	**Visual stimuli patterns**	**N**	**Mean**	**Std. deviation**	**Minimum**	**Maximum**
Simple patterns	P25-25-25	22	4.95	2.578	2	10
	P30-30-30	22	4.29	2.591	1	10
	P35-35-35	22	2.95	2.459	1	10
Rhythmic patterns	P25-30-35	22	2.90	2.278	0	7
	P30-25-35	22	4.57	2.712	1	10
	P25-35-30	22	3.48	2.600	0	9
	P30-35-25	22	3.76	2.548	0	9
	P35-25-30	22	4.81	2.657	1	10
	P35-30-25	22	4.29	2.101	1	9

*N, Number of subjects; Mean, Average fatigue rate for all subjects in each pattern; Std. Deviation, Fatigue rate standard deviation in 22 subjects respect to each pattern; Minimum, min fatigue rate for each pattern; Maximum, maximum fatigue rate for each pattern*.

**Table 8:** *Post hoc* analysis results: Results of fatigue rate comparison between each pair of visual stimuli patterns.

**Other Patterns Pattern**	**P30-30-30**	**P35-35-35**	**P25-30-35**	**P25-35-30**	**P30-25-35**	**P30-35-25**	**P35-25-30**	**P35-30-25**
P25-25-25	*Z* = 0.955, *P* = 0.339	***Z*** = −**3.541**, ***P*** < 0.001^*^	**Z** = −**3.202**, ***P*** < 0.001^**^	***Z*** = −**3.443**, ***P*** < 0.001^**^	*Z* = −1.504, *P* = 0.132	*Z* = −2.704, *P* = 0.007	*Z* = −0.792, *P* = 0.428	*Z* = −1.807, *P* = 0.071
P30-30-30		***Z*** = −**3.220**, ***P*** < 0.001^*^	*Z* = −3.050, *P* = 0.002	*Z* = −1.802, *P* = 0.072	*Z* = −0.066, *P* = 0.947	*Z* = −1.551, *P* = 0.121	*Z* = −0.754, *P* = 0.455	*Z* = −0.209, *P* = 0.835
P35-35-35			*Z* = −0.124, *P* = 0.902	*Z* = −1.506, *P* = 0.132	*Z* = −2.967, *P* = 0.003	*Z* = −1.283, *P* = 0.199	***Z*** = −**3.404**, ***P*** < 0.001^**^	*Z* = −2.441, *P* = 0.015
P25-30-35				*Z* = −1.592, *P* = 0.111	*Z* = −2.917, *P* = 0.004	*Z* = −2.419, *P* = 0.016	*Z* = −3.095, *P* = 0.002	*Z* = −2.393, *P* = 0.017
P25-35-30					***Z*** = −**3.206**, ***P*** < 0.001^***^	*Z* = −0.296, *P* = 0.768	***Z*** = −**3.447**, ***P*** < 0.001^***^	*Z* = −1.947, *P* = 0.052
P30-25-35						*Z* = −2.107, *P* = 0.035	*Z* = −1.209, *P* = 0.227	*Z* = −0.532, *P* = 0.595
P30-35-25							*Z* = −2.378, *P* = 0.017	*Z* = −1.909, *P* = 0.056
P35-25-30								*Z* = −1.119, *P* = 0.263

* *Significant fatigue rate changes in pair of simple group patterns comparison*.

** *Significant fatigue rate changes when one pattern in simple group compared with one pattern in rhythmic group*.

*** *Significant fatigue rate changes in pair of rhythmic group patterns comparison*.

*Bold values are statistically significant*.

## Discussion

In this study, we have used high-frequency low THD sine wave with simple and rhythmic patterns and presented them as visual stimuli. Additionally, we analyzed SSVEP responses with three well known methods (PSD, CCA and LASSO) in order to discriminate patterns. We also evaluated the effects of simple and rhythmic patterns on subjective fatigue rate in normal subjects. We found that the rhythmic patterns had significantly higher accuracy rates in single trial and overall insignificant higher rates in the mean of 10 trials, and resulted in comparatively lesser though insignificant subjective fatigue.

In comparison to the two apparently similar studies that didn't examine subjective fatigue rate and have utilized square pulse shape stimuli type without reporting THD rate (Zhang et al., 2012b, 2015), we used sine shaped high frequency range stimuli and also examined the simple and rhythmic group accuracies as well as their subjective fatigue rate (see Supplementary Table S4 for detailed comparison).

Most SSVEP studies have utilized low-frequency visual stimuli range that yields high SNR results and resultant high accuracy achievement, mostly higher than 80% (Zhang et al., 2012b; Sakurada et al., 2013). However, at lower frequency ranges (~8 Hz), the risk of photosensitive epileptic seizures and high fatigue rate have to be considered. These two main challenges encouraged researchers to carry out SSVEP studies at high frequency range. At high frequency range, because of comparatively lower SNR, accuracy obtained is also lower (between 70 and 85%) than that of low-frequency studies (Garcia, 2008). We have attempted to improve the accuracy of high frequency stimuli by examining the rhythmic patterns and compared them with simple ones.

### Validation of the rhythmic visual stimuli patterns

In our study, six permutations were designed with selection of three different high frequencies (25, 30, and 35 Hz) in 3-sequence including ascending, descending and zigzag rhythms (Figures 2, 3, and Supplementary Tables S2, S3). Three simple patterns had same frequency in the sequence.

Comparison of simple and rhythmic patterns showed higher accuracy rate for rhythmic patterns group (Table 6 and see Supplementary Table S5). Recently a high-frequency SSVEP study has reported accuracy rates of 90–100% employing improved design paradigm and use of synchronized averaging of EEG trials (Zhang et al., 2015). We designed visual stimulus patterns at high-frequency range and obtained similar results (>90%) (Tables 1, 2). Our results for single trials analysis are in the range of 73–74% (Tables 4, 5) and are comparable with (Dreyer et al., 2017) that reported 33–87%. Our single trial accuracy rate was also comparable with a recent low frequency study (Zhang et al., 2012b). Additionally, our mean of 10 trials accuracy rates for rhythmic and simple groups are better than another high frequency SSVEP study (Zhang et al., 2015; see Supplementary Table S4). Use of appropriate sequence coding increases the unpredictability of visual patterns and leads to discriminative SSVEP response. Statistical analysis confirmed the discrimination of patterns by examination of the amplitude of SSVEP features. As shown in Supplementary Tables S2, S3, simple, ascending, descending and zigzag patterns coefficient vectors had significant differences with each other (see Supplementary Figure S5). This is also seen in different patterns illustrated by CCA coefficients matrix (see Supplementary Figures S3, S5).

### PSD, CCA, and LASSO results comparison

Most SSVEP studies have used PSD based method for SSVEP recognition and so we first tried to analyze data with this method. For 2 s rectangular window mean accuracy rate was obtained to be equal to 88.35%. However, at high-frequency range, obviously, this method was not what we expected because of low amplitude in frequency spectrum of SSVEP response compared to the low-frequency range (Garcia, 2008). CCA and LASSO methods could improve SSVEP-BCI speed compared to PSD. By utilizing these two methods, more robust discriminative features in shorter TW of EEG data can be extracted (Zhang et al., 2012a). For sequence coding to design the visual stimulus patterns, it was important to analyze the data in shorter TWs. As shown in the CCA and LASSO results (Figure 8), for *TW* = 0.75 s, we were able to obtain acceptable accuracy rate of higher than 90%. Our results of comparison between CCA and LASSO at different TWs were not significant. However, at low TWs length (≤1.25 s), LASSO did show better performance compared to CCA while at *TW* > 1.25 s, CCA showed better results than LASSO. These results are consistent with a previous study that reported better performance for LASSO at low TWs duration [0.5–2.5 s], while for *TW* > 2.5 s, CCA had same and even better results (Zhang et al., 2012a). However, compared to our study, they used low frequency, square shaped visual patterns stimuli and had 9 participants (our study had 22 participants).

Our CCA and LASSO results point to the importance of shape of visual stimulus. Based on the knowledge of authors, studies carried out so far that have compared LASSO and CCA analysis of SSVEP, have not considered stimulus harmonic distortion (Zhang et al., 2012a,b). These and other studies have used square pulse shape stimulus and therefore the odd harmonics of fundamental frequency existed in their visual stimulus frequency spectrum. Thus, the SSVEP response that was recognized in these studies did not correspond only to the fundamental frequency. In our study, by designing the sine shape visual patterns with low THD it was possible to have SSVEP response elicited by fundamental frequency almost devoid of distortion. Thus, our CCA and LASSO analysis of the SSVEP responses were largely not affected by the odd harmonics of fundamental frequency of the input stimuli.

### Fatigue rate evaluation

Using high frequency range stimuli can reduce the subjective fatigue rate compared to the low frequency (Diez et al., 2011). Studies that have examined the fatigue rate included simple high frequency (Diez et al., 2011, 2013) while others have used the advantages of high frequency range as carrier signal (Chang et al., 2014; Dreyer and Herrmann, 2015; Dreyer et al., 2017). This was done to decrease visual perceptibility and consequently decrease the fatigue rate. In our study fatigue rate was evaluated for the high frequency rhythmic and simple patterns.

We used VAS as psychometric response scale for fatigue rate evaluation. Our results show that there was overall no significant difference between subjective fatigue rates of simple and rhythmic stimuli pattern groups, though the VAS was lower for the rhythmic patterns group. While significant fatigue rate change was obtained within both simple and rhythmic visual stimuli groups, however, within group fatigue rate variations in the rhythmic group patterns were lower than the simple group. In addition, rhythmic group pattern P25-30-35 had the least VAS score of our study among all the individual patterns. Maximum and minimum fatigue rates were observed for P25-25-25 and P35-35-35 patterns respectively, in the simple group, indicating that higher frequencies cause reduced subjective fatigue. Maximum and minimum fatigue scores in the rhythmic group was for P35-25-30 and P25-30-35 patterns respectively, indicating the importance of the order of frequencies presented in a sequence (see Supplementary Figure S6).

In this study, we observed higher but insignificant trend in VAS values over time indicating higher subjective fatigue from beginning to the end of experiment (Supplementary Figures S6, S7). As subjective fatigue was expected to increase over time in our experiment, compared to few other recent SSVEP studies (Diez et al., 2011, 2013; Dreyer and Herrmann, 2015; Dreyer et al., 2017) we kept acceptable rest time (2 min) to minimize it. However, the aim of our study was not to evaluate fatigue at various time points for each pattern, rather we were more interested in knowing the fatigue rate between simple and rhythmic groups as well as its variation within these two groups. Thus, three simple group patterns were randomly interspersed within six rhythmic group patterns. This order was kept same for all the subjects so as to get authentic data. As a result, the patterns of rhythmic group were distributed in the first, middle and in the last time points of each experiment with simple group in between them. Overall, this minimized the influence of time in VAS analysis for simple and rhythmic groups. The duration of our experiment (32 min) might be seen as longer, as seen by higher VAS scores with time, however, evaluation of fatigue between consecutive patterns was not aimed in our study. We suggest future experiments to evaluate the fatigue rate with individual patterns.

### Limitations of the study and suggestions for the future works

For designing visual stimulus patterns, several limitations have to be considered. Perhaps the most important point is the number of frequencies that are selected. Although, there is a trade-off between accuracy and speed of BCI applications which is related to the length of each trial, as when the number of frequencies in each sequence increases, the speed of the BCI decreases. However, still, a high accuracy rate can be obtained. In this study, we allocated equal time for each frequency in one trial. However, for future works, it is suggested that time of each frequency can be optimized and set differently for improving the speed. Additionally, for increasing the speed, it could be better to detect the lowest number of each frequency repetition for SSVEP generation and then setting the least time required for placing each frequency in the specific pattern. For example, higher frequencies may need lesser time compared to the lower frequencies, because in Ti duration time, the number of periods that higher frequencies are repeated is more than lower frequencies. Thus, SSVEP response to higher frequencies may be generated faster than low frequencies.

For N individual pattern analysis, we suggest that the order of patterns should be randomized and selected from existing N! permutations. However, this approach will obviously require more time and experiments for evaluation of the all permutations. In addition, it will increase the subjective fatigue rate over time and may lead to significant effect on the responses. Finally, within/between subject effects and responses should also be examined.

For the analysis of SSVEP, various methods of analysis are reported recently (Liu et al., 2014; Cao et al., 2015; Zhang et al., 2015). We only used three conventional methods (CCA, LASSO and PSD) for analysis of SSVEP signals. In future works, these as well as other recent methods can be used.

In a real BCI application, various patterns are applied simultaneously. Therefore, for designing an online synchronous or even asynchronous real BCI based on employed rhythmic patterns, appropriate modifications in hardware set up must be considered together with the selection of accurate frequency value.

VAS measurement tool was used for subjective fatigue rate evaluation in our study. It's important to quantify fatigue rate more accurately and not only based on spectral analysis (Cao et al., 2014). For future studies, using an objective method for fatigue evaluation will be more valuable for consistency between SSVEP-BCI applications. For fatigue rate reduction, our suggestion is to use different colors along with rhythmic patterns as this leads to significant effect on SSVEP responses (Duszyk et al., 2014).

## Conclusion

High frequency sine shaped rhythmic and simple patterns had low THD, and higher and similar accuracy rates. Rhythmic stimuli patterns showed insignificantly lower fatigue rate than simple patterns. We conclude that both rhythmic and simple visual high frequency sine wave stimuli can provide a base for further human subject SSVEP based BCI studies.

## Author contributions

AK did experiments, acquired and analyzed the data, wrote first draft of manuscript. ZS acquired and analyzed data, helped in writing manuscript. MF designed the hardware and setup for the experiment. ES acquired and analyzed data, helped in writing manuscript. MH experimental design and data interpretation, Co-supervisor. AM Project Co-director, Co-supervisor, data interpretation, designed the hardware and setup for the experiment. BM Co-supervisor, data interpretation, designed the hardware for the experiment. AJ Original idea for study, Project Director, Principal Investigator, and Supervisor.

### Conflict of interest statement

The authors declare that the research was conducted in the absence of any commercial or financial relationships that could be construed as a potential conflict of interest.
