# Supplementary material for: Use of Sine Shaped High-Frequency Rhythmic Visual Stimuli Patterns for SSVEP Response Analysis and Fatigue Rate Evaluation in Normal Subjects
Source: Front Hum Neurosci. 2018 May 28;12:201. doi: 10.3389/fnhum.2018.00201 (PMC5985331; doi:10.3389/fnhum.2018.00201)
Supplement: Supplementary file 1 [file Table_1.DOCX]

**Supplementary table S1**: **The order of presented visual stimuli patterns for 22 subjects.**

| **The order of presented patterns** | **Patterns** |
| --- | --- |
| 1 | P25-30-35 |
| 2 | P30-35-25 |
| 3 | P25-35-30 |
| 4 | P35-35-35 |
| 5 | P35-30-25 |
| 6 | P25-25-25 |
| 7 | P30-30-30 |
| 8 | P35-25-30 |
| 9 | P30-25-35 |
