# Supplementary material for: Use of Sine Shaped High-Frequency Rhythmic Visual Stimuli Patterns for SSVEP Response Analysis and Fatigue Rate Evaluation in Normal Subjects
Source: Front Hum Neurosci. 2018 May 28;12:201. doi: 10.3389/fnhum.2018.00201 (PMC5985331; doi:10.3389/fnhum.2018.00201)
Supplement: Supplementary file 2 [file Table_2.DOCX]

**Supplementary table S2:** **Matrix of CCA coefficients for nine patterns.**

| **Patterns** | **Mean amplitude R-first (SD)** | **Mean amplitude R-second (SD)** | **Mean amplitude R-third (SD)** |
| --- | --- | --- | --- |
| P25-25-25 (Simple) | 0.1822 (0.08) | 0.1791 (0.08) | 0.1727 (0.07) |
| P30-30-30 (Simple) | 0.1407 (0.06) | 0.1581 (0.08) | 0.1554 (0.06) |
| P35-35-35 (Simple) | 0.1306 (0.05) | 0.1328 (0.06) | 0.1354 (0.06) |
| P25-30-35 (Ascending) | 0.1827 (0.07) | 0.1546 (0.06) | 0.1190 (0.05) |
| P25-35-30 (Zigzag) | 0.1827 (0.07) | 0.1200 (0.05) | 0.1478 (0.07) |
| P30-25-35 (Zigzag) | 0.1478 (0.07) | 0.1899 (0.08) | 0.1190 (0.05) |
| P30-35-25(Zigzag) | 0.1478 (0.07) | 0.1200 (0.05) | 0.1869 (0.08) |
| P35-25-30 (Zigzag) | 0.1265 (0.05) | 0.1899 (0.08) | 0.1478 (0.07) |
| P35-30-25 (Descending) | 0.1265 (0.05) | 0.1546 (0.06) | 0.1869 (0.08) |
