# Supplementary material for: Use of Sine Shaped High-Frequency Rhythmic Visual Stimuli Patterns for SSVEP Response Analysis and Fatigue Rate Evaluation in Normal Subjects
Source: Front Hum Neurosci. 2018 May 28;12:201. doi: 10.3389/fnhum.2018.00201 (PMC5985331; doi:10.3389/fnhum.2018.00201)
Supplement: Supplementary file 3 [file Table_3.DOCX]

**Supplementary table S3: Matrix of LASSO coefficients for nine patterns.**

| **Patterns** | **Mean amplitude CD-First (SD)** | **Mean amplitude CD-second (SD)** | **Mean amplitude CD-third (SD)** |
| --- | --- | --- | --- |
| P25-25-25 (Simple) | 3.14 (1.40) | 3.23 (1.53) | 3.00 (1.39) |
| P30-30-30 (Simple) | 3.03 (1.32) | 3.46 (1.88) | 3.39 (1.63) |
| P35-35-35 (Simple) | 2.56 (1.17) | 2.79 (1.36) | 2.82 (1.41) |
| P25-30-35 (Ascending) | 3.157 (1.43) | 3.35 (1.45) | 2.48 (1.13) |
| P25-35-30 (Zigzag) | 3.157 (1.43) | 2.64 (1.35) | 3.18 (1.73) |
| P30-25-35 (Zigzag) | 3.05(1.32) | 3.20 (1.62) | 2.48 (1.13) |
| P30-35-25 (Zigzag) | 3.05 (1.32) | 2.64 (1.35) | 3.00 (1.41) |
| P35-25-30 (Zigzag) | 2.53 (1.05) | 3.20 (1.62) | 3.18 (1.73) |
| P35-30-25 (Descending) | 2.53 (1.05) | 3.35 (1.45) | 3.00 (1.41) |
