# Supplementary material for: Use of Sine Shaped High-Frequency Rhythmic Visual Stimuli Patterns for SSVEP Response Analysis and Fatigue Rate Evaluation in Normal Subjects
Source: Front Hum Neurosci. 2018 May 28;12:201. doi: 10.3389/fnhum.2018.00201 (PMC5985331; doi:10.3389/fnhum.2018.00201)
Supplement: Supplementary file 4 [file Table_4.DOCX]

| **Study Parameters** | **Zhang et.al 2012** | **Zhang et.al 2015** | | | **Our study** | |
| --- | --- | --- | --- | --- | --- | --- |
| **Sample size** | **10 males** | **3 males** | | | **22 (11 females and 11 males)** | |
| **Frequency range** | **Low (7.5, 12 Hz)** | **High (25, 33.33, 40 Hz)** | | | **High (25, 30, 35 Hz)** | |
| **Stimuli shape** | **Square** | **Square** | | | **Sine** | |
| **Screen or monitor type** | **LCD** | **CRT** | | | **LED** | |
| **Fatigue rate evaluation** | **No** | **No** | | | **Yes** | |
| **THD rate report** | **No** | **No** | | | **Lower than 0.1%** | |
| **Accuracy rate (%)**  **WL=2 Sec.** | **Single trial** | **# Trials** | **Rhythmic** | **Simple** | **Rhythmic** | **Simple** |
|  | **97.75**  **(The study didn't separate rhythmic and simple patterns)** | **Single trial** | **-** | **-** | **76.24(16.34)^*^**  **75.40(15.23)^**^** | **71.34(17.09)^*^**  **70.01(16.37)^**^** |
|  |  | **Mean of 10 trials** | **92** | **70** | **100(0)^*^**  **99.24(3.55)^**^** | **98.48(7.10)^*^**  **98.48(7.10)^**^** |

**Supplementary table S4: Comparison of our low THD rate with sine wave stimuli with the two apparently similar studies that used square pulse with high THD rate. Additionally, main parameters are compared for overview of the novelty of our study. \**

*CCA analysis results

**LASSO analysis results
