# Supplementary material for: Use of Sine Shaped High-Frequency Rhythmic Visual Stimuli Patterns for SSVEP Response Analysis and Fatigue Rate Evaluation in Normal Subjects
Source: Front Hum Neurosci. 2018 May 28;12:201. doi: 10.3389/fnhum.2018.00201 (PMC5985331; doi:10.3389/fnhum.2018.00201)
Supplement: Supplementary file 5 [file Table_5.DOCX]

**Supplementary table S5: Rhythmic and Simple group results comparison for CCA and LASSO.**

| **Subjects**  **ID** | **CCA accuracy (%) in WL=2 Sec.** | | | | **LASSO accuracy (%) in WL=2 Sec.** | | | |
| --- | --- | --- | --- | --- | --- | --- | --- | --- |
|  | **Single trial** | | **Mean of 10 trials** | | **Single trial** | | **Mean of 10 trials** | |
|  | **Simple** | **Rhythmic** | **Simple** | **Rhythmic** | **Simple** | **Rhythmic** | **simple** | **Rhythmic** |
| S1 | 34.88 | 40.89 | 100 | 100 | 49.35 | 58.11 | 100 | 100 |
| S2 | 64.88 | 68.67 | 66.67 | 100 | 61.58 | 69.22 | 66.67 | 100 |
| S3 | 89.32 | 98.67 | 100 | 100 | 88.24 | 97.00 | 100 | 100 |
| S4 | 92.66 | 96.44 | 100 | 100 | 93.80 | 98.11 | 100 | 100 |
| S5 | 65.99 | 64.78 | 100 | 100 | 67.13 | 62.00 | 100 | 100 |
| S6 | 84.88 | 87.56 | 100 | 100 | 87.13 | 85.89 | 100 | 100 |
| S7 | 80.43 | 79.22 | 100 | 100 | 74.91 | 78.11 | 100 | 100 |
| S8 | 62.66 | 74.78 | 100 | 100 | 57.13 | 73.11 | 100 | 100 |
| S9 | 90.43 | 93.67 | 100 | 100 | 86.02 | 90.33 | 100 | 100 |
| S10 | 79.32 | 89.22 | 100 | 100 | 80.46 | 88.11 | 100 | 100 |
| S11 | 85.99 | 89.78 | 100 | 100 | 86.02 | 89.78 | 100 | 100 |
| S12 | 47.10 | 49.78 | 100 | 100 | 40.46 | 48.11 | 100 | 83.33 |
| S13 | 94.88 | 94.22 | 100 | 100 | 94.91 | 95.89 | 100 | 100 |
| S14 | 77.10 | 82.56 | 100 | 100 | 74.91 | 80.89 | 100 | 100 |
| S15 | 50.43 | 51.44 | 100 | 100 | 51.58 | 51.44 | 100 | 100 |
| S16 | 58.21 | 66.44 | 100 | 100 | 57.13 | 68.11 | 100 | 100 |
| S17 | 77.10 | 83.67 | 100 | 100 | 76.02 | 84.78 | 100 | 100 |
| S18 | 54.88 | 64.22 | 100 | 100 | 53.80 | 61.44 | 100 | 100 |
| S19 | 49.32 | 58.67 | 100 | 100 | 43.80 | 56.44 | 100 | 100 |
| S20 | 83.77 | 85.89 | 100 | 100 | 77.13 | 83.11 | 100 | 100 |
| S21 | 61.55 | 72.00 | 100 | 100 | 63.80 | 63.67 | 100 | 100 |
| S22 | 83.77 | 84.78 | 100 | 100 | 74.91 | 75.33 | 100 | 100 |
| **Mean(SD)** | **71.34(17.09)** | **76.24(16.37)** | **98.48(7.10)** | **100(0.00)** | **70.01(16.3)** | **75.40(15.23)** | **98.48(7.10)** | **99.24(3.55)** |
