# Supplementary material for: Use of Sine Shaped High-Frequency Rhythmic Visual Stimuli Patterns for SSVEP Response Analysis and Fatigue Rate Evaluation in Normal Subjects
Source: Front Hum Neurosci. 2018 May 28;12:201. doi: 10.3389/fnhum.2018.00201 (PMC5985331; doi:10.3389/fnhum.2018.00201)
Supplement: Supplementary file 6 [file Image_1.PDF]

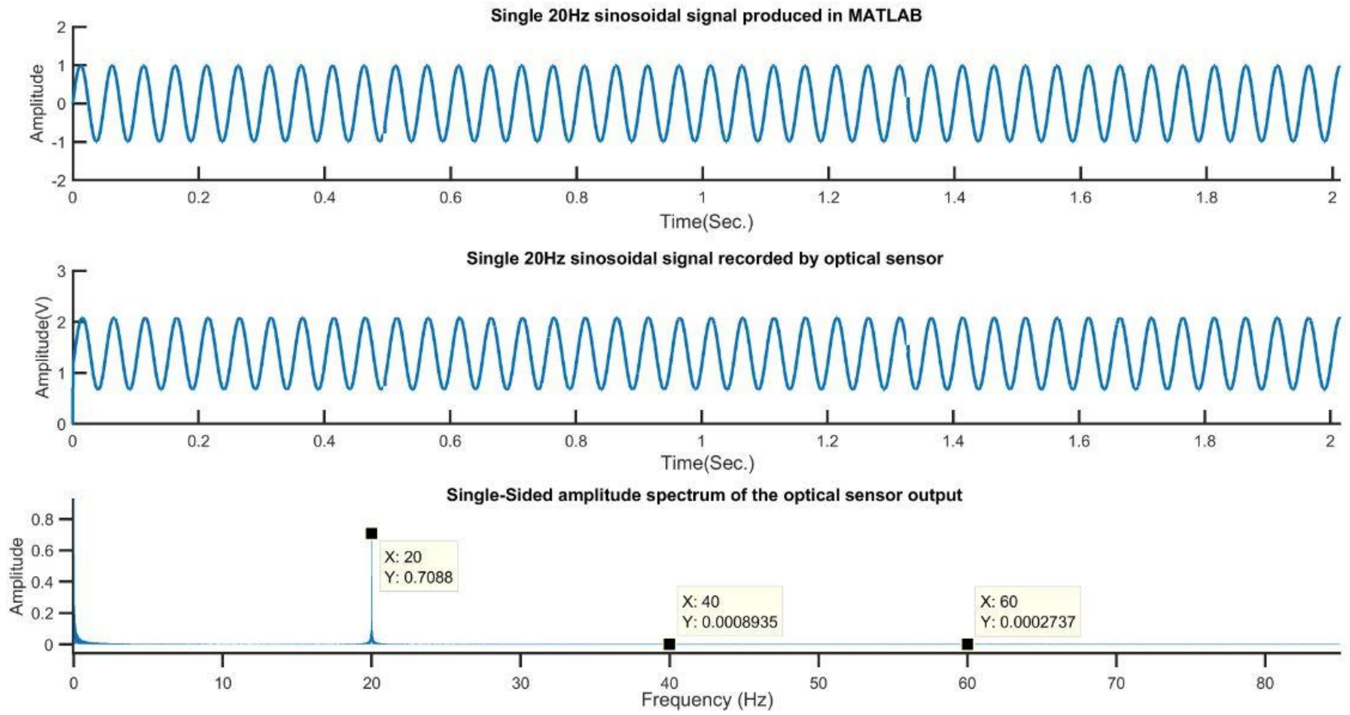

Supplementary figure S1: Optical sensor response to a pure sine 20 Hz signal applied to the LED with precise driver. Top: Pure 20 Hz sinusoidal signal applied to the LED. Middle: Pure sine 20 Hz signal recorded by optical sensor Buttom: Frequency response of the optical sensor output.

Total harmonic distortion (THD) can measured by the following formula:

$$THD_{Luminance} \cong \frac{\sqrt{H_2^2 + H_3^2}}{H_1} = \frac{\sqrt{0.00089^2 + 0.00027^2}}{0.70} = 0.001 = 0.1\%$$

Where H_2 and H_3 are the amplitudes of second and third harmonic respectively and H_1 is the amplitude of the fundamental frequency.
