# Supplementary material for: Use of Sine Shaped High-Frequency Rhythmic Visual Stimuli Patterns for SSVEP Response Analysis and Fatigue Rate Evaluation in Normal Subjects
Source: Front Hum Neurosci. 2018 May 28;12:201. doi: 10.3389/fnhum.2018.00201 (PMC5985331; doi:10.3389/fnhum.2018.00201)
Supplement: Supplementary file 7 [file Image_2.PDF]

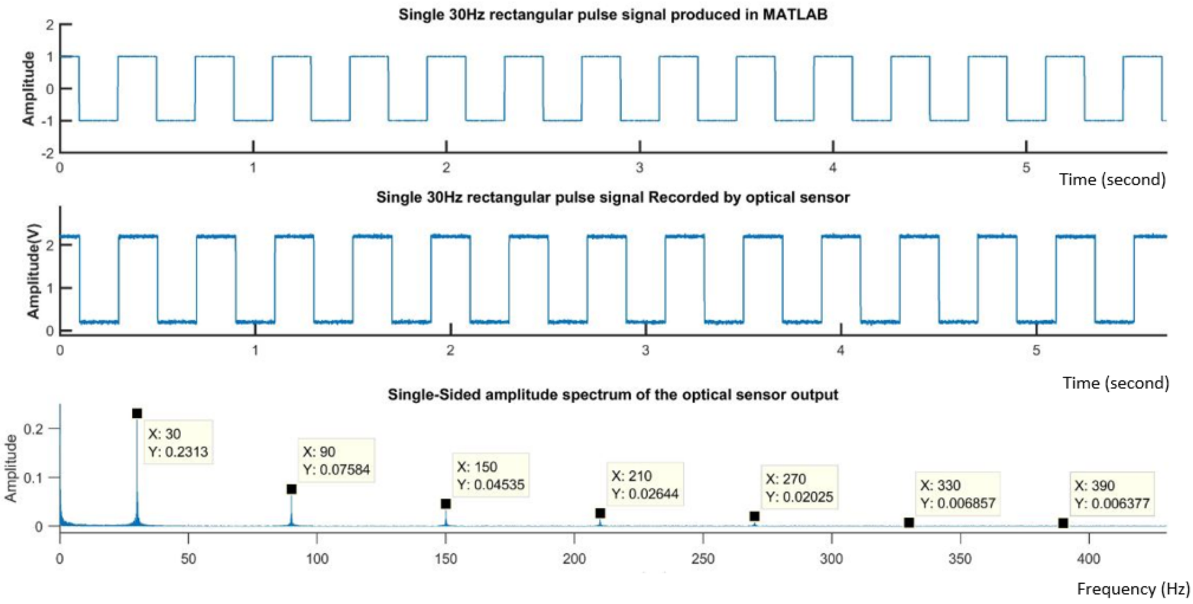

Supplementary figure S2: Optical sensor response to a square pulse 30 Hz signal applied to the LED with precise driver. Top: square pulse 30 Hz signal applied to the LED. Middle: Square pulse 30 Hz signal recorded by optical sensor Buttom: Frequency response of the optical sensor.
