# Supplementary material for: Use of Sine Shaped High-Frequency Rhythmic Visual Stimuli Patterns for SSVEP Response Analysis and Fatigue Rate Evaluation in Normal Subjects
Source: Front Hum Neurosci. 2018 May 28;12:201. doi: 10.3389/fnhum.2018.00201 (PMC5985331; doi:10.3389/fnhum.2018.00201)
Supplement: Supplementary file 8 [file Image_3.PDF]

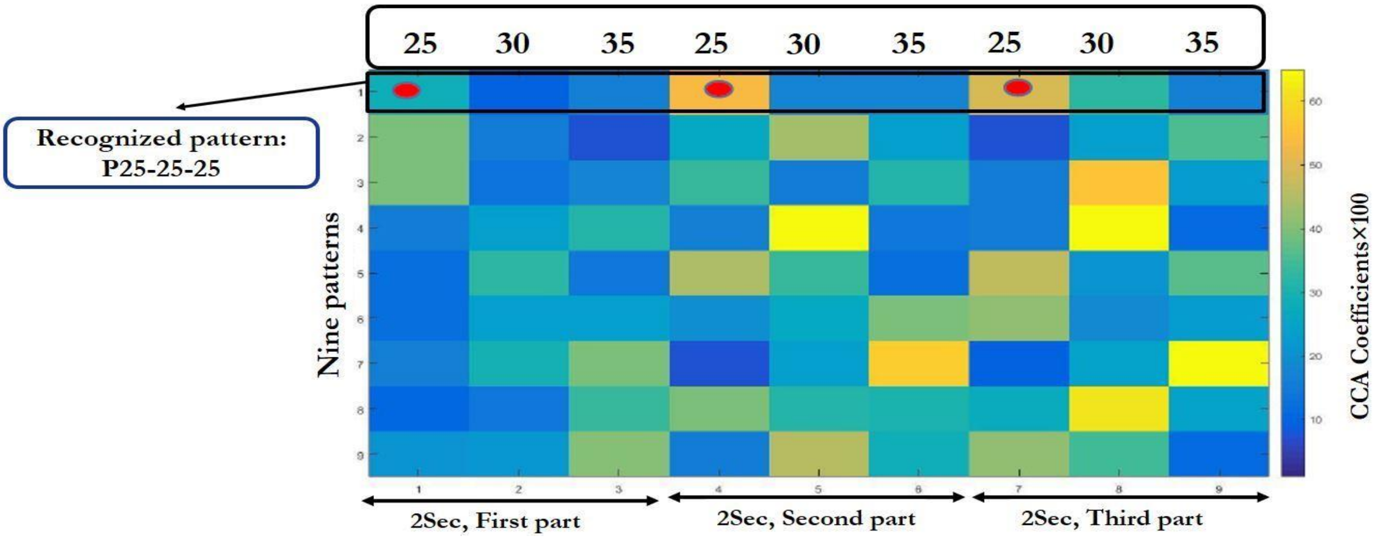

Supplementary figure S3: Targeted pattern recognition for single subject according the coefficient matrix and output of the classifier. For P25-25-25, the classifier determined the pattern correctly. For each row all the 9 patterns are labeled as 0 and 1 and then accuracy was calculated according to Eq11.
