# Supplementary material for: Use of Sine Shaped High-Frequency Rhythmic Visual Stimuli Patterns for SSVEP Response Analysis and Fatigue Rate Evaluation in Normal Subjects
Source: Front Hum Neurosci. 2018 May 28;12:201. doi: 10.3389/fnhum.2018.00201 (PMC5985331; doi:10.3389/fnhum.2018.00201)
Supplement: Supplementary file 9 [file Image_4.PDF]

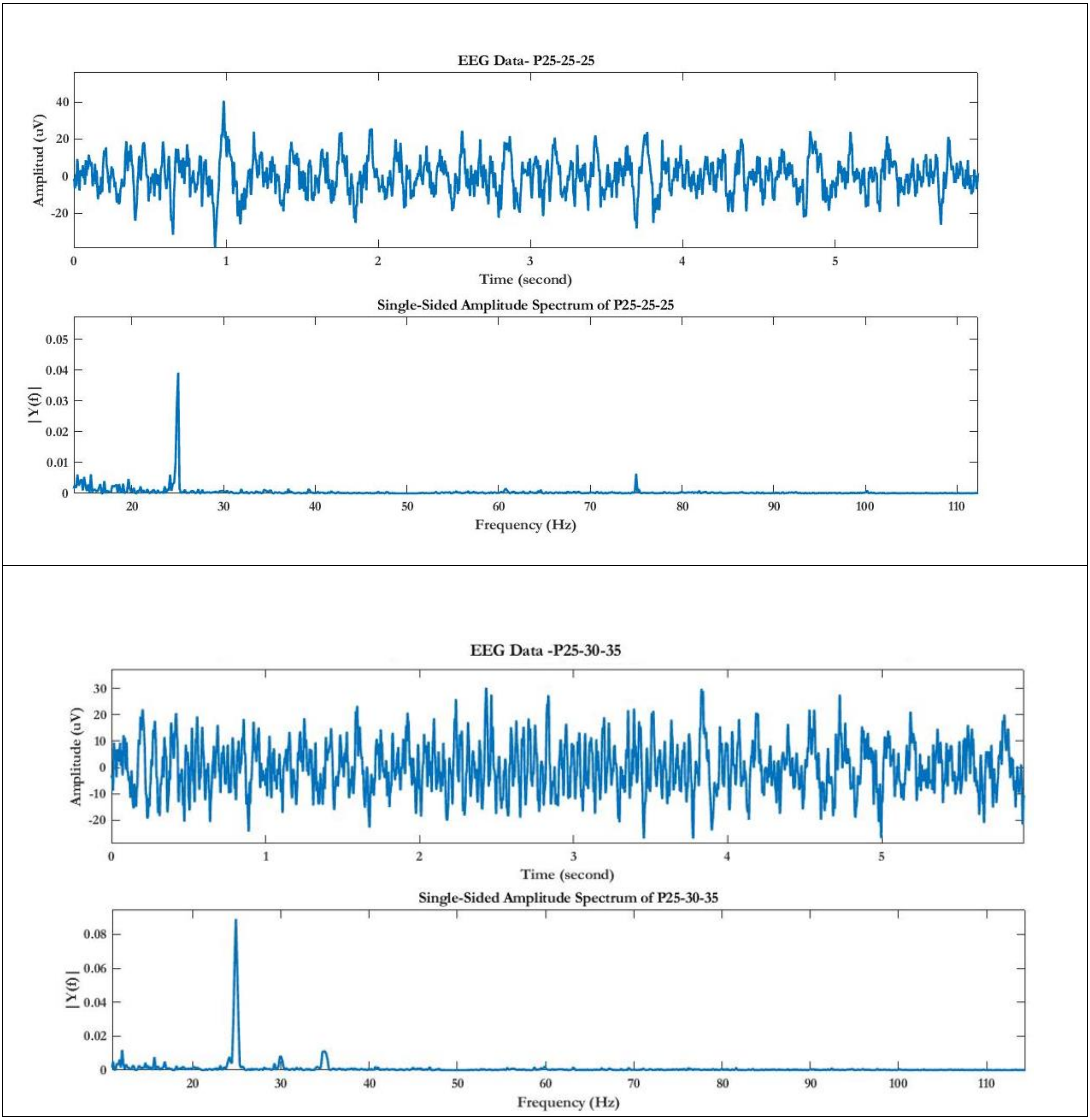


**Supplementary figure S4: Grand average of the nine SSVEP patterns: Top plot: EEG data in time domain, Bottom: Power spectral density of SSVEP responses.**

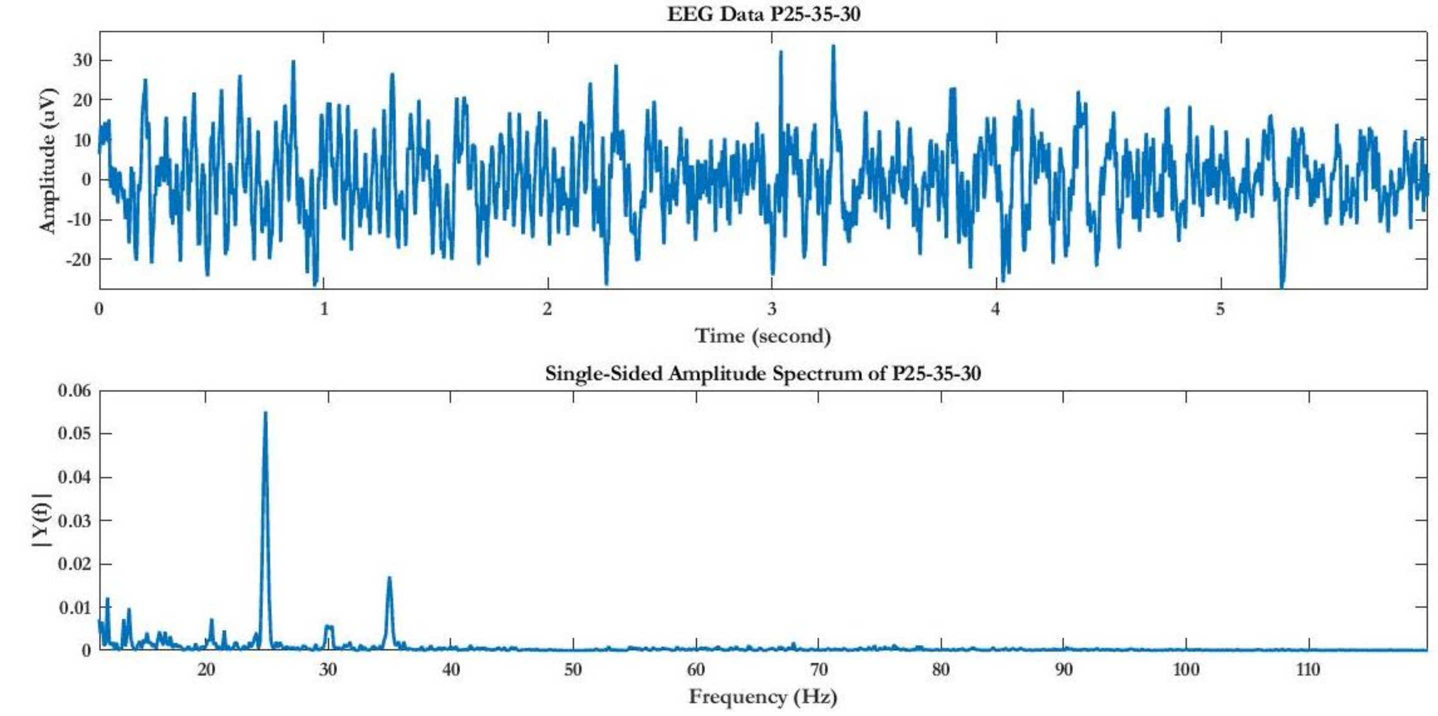
EEG Data P25-35-30
Amplitude (uV)
Time (second)
Single-Sided Amplitude Spectrum of P25-35-30
|Y(f)|
Frequency (Hz)

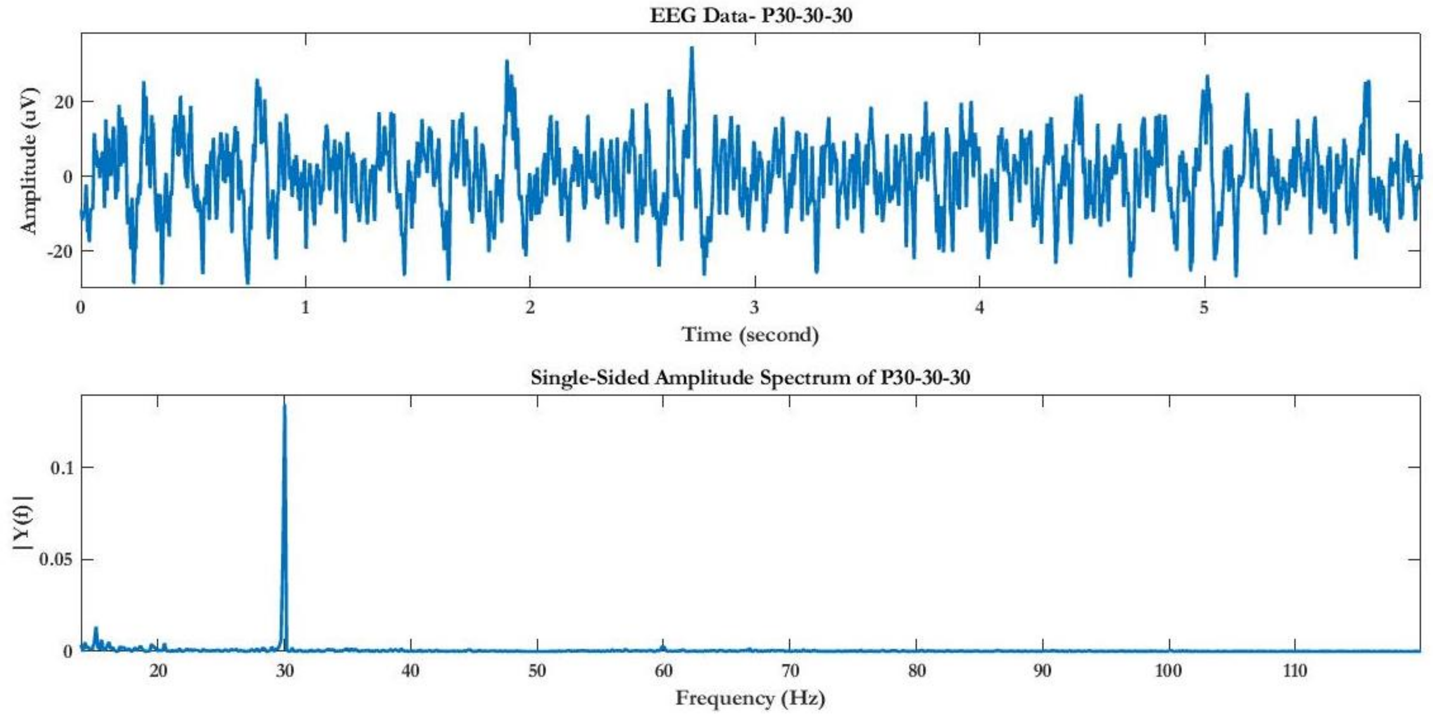
EEG Data- P30-30-30
Amplitude (uV)
Time (second)
Single-Sided Amplitude Spectrum of P30-30-30
|Y(f)|
Frequency (Hz)

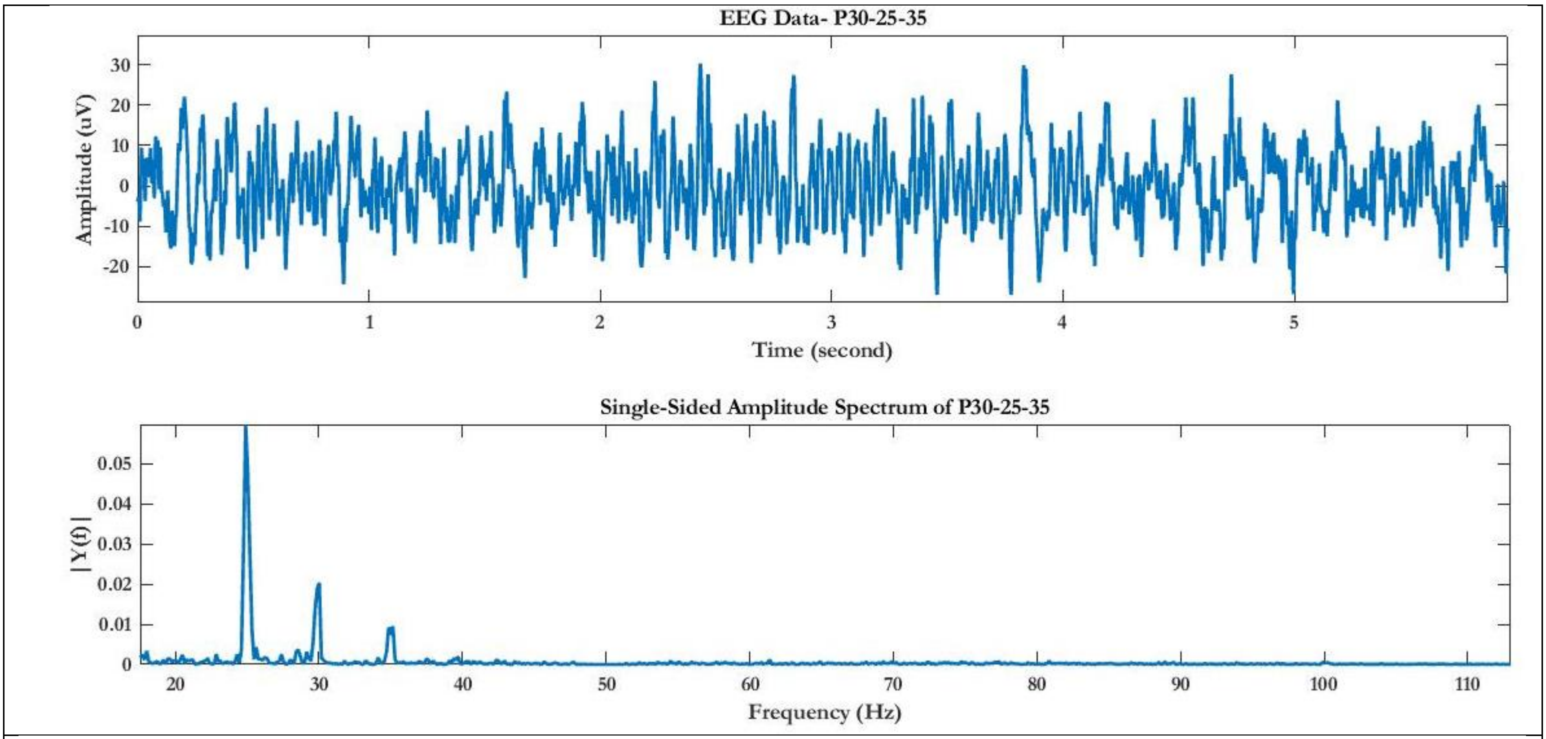
EEG Data- P30-25-35
Amplitude (uV)
Time (second)
Single-Sided Amplitude Spectrum of P30-25-35
|Y(f)|
Frequency (Hz)

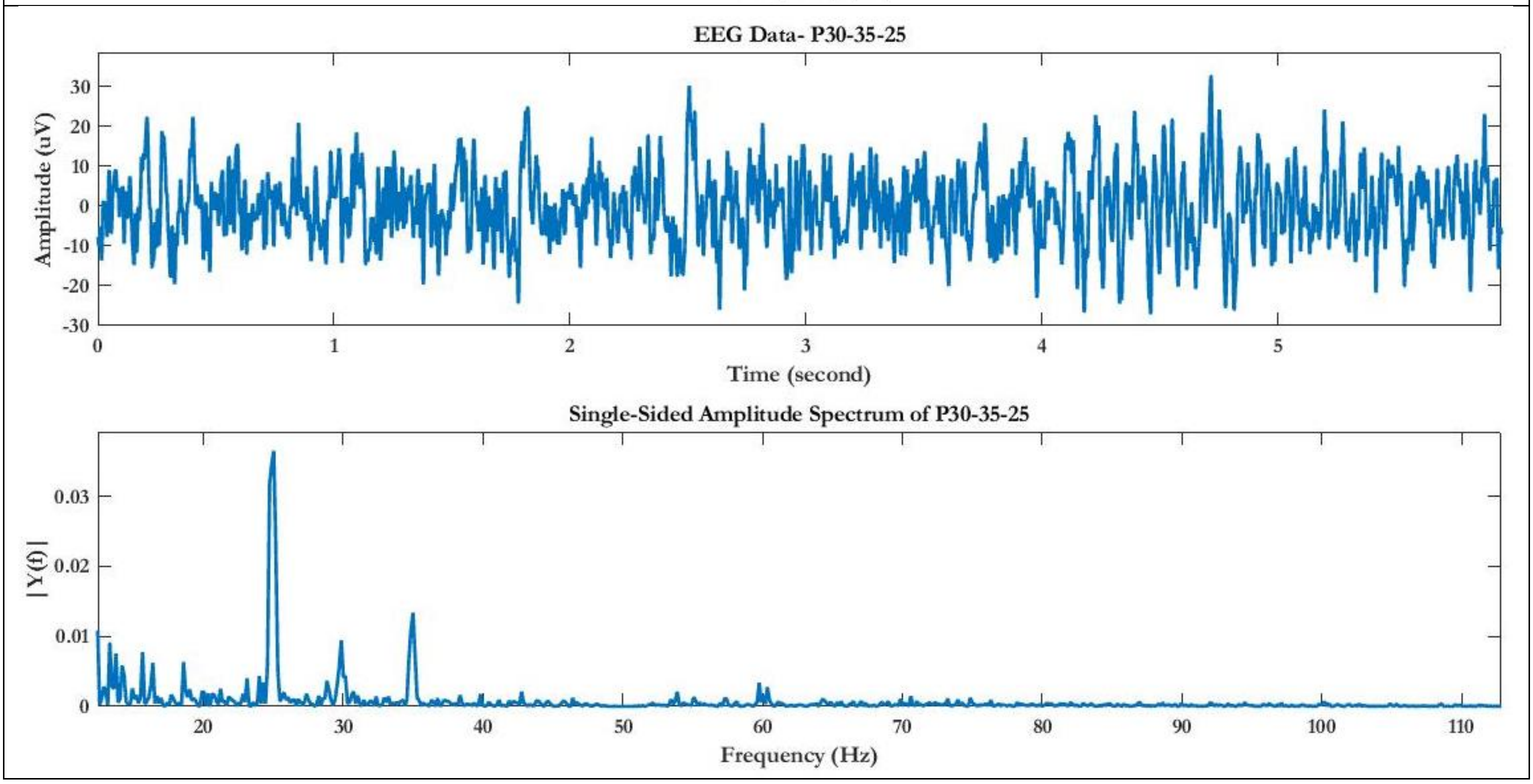
EEG Data- P30-35-25
Amplitude (uV)
Time (second)
Single-Sided Amplitude Spectrum of P30-35-25
|Y(f)|
Frequency (Hz)

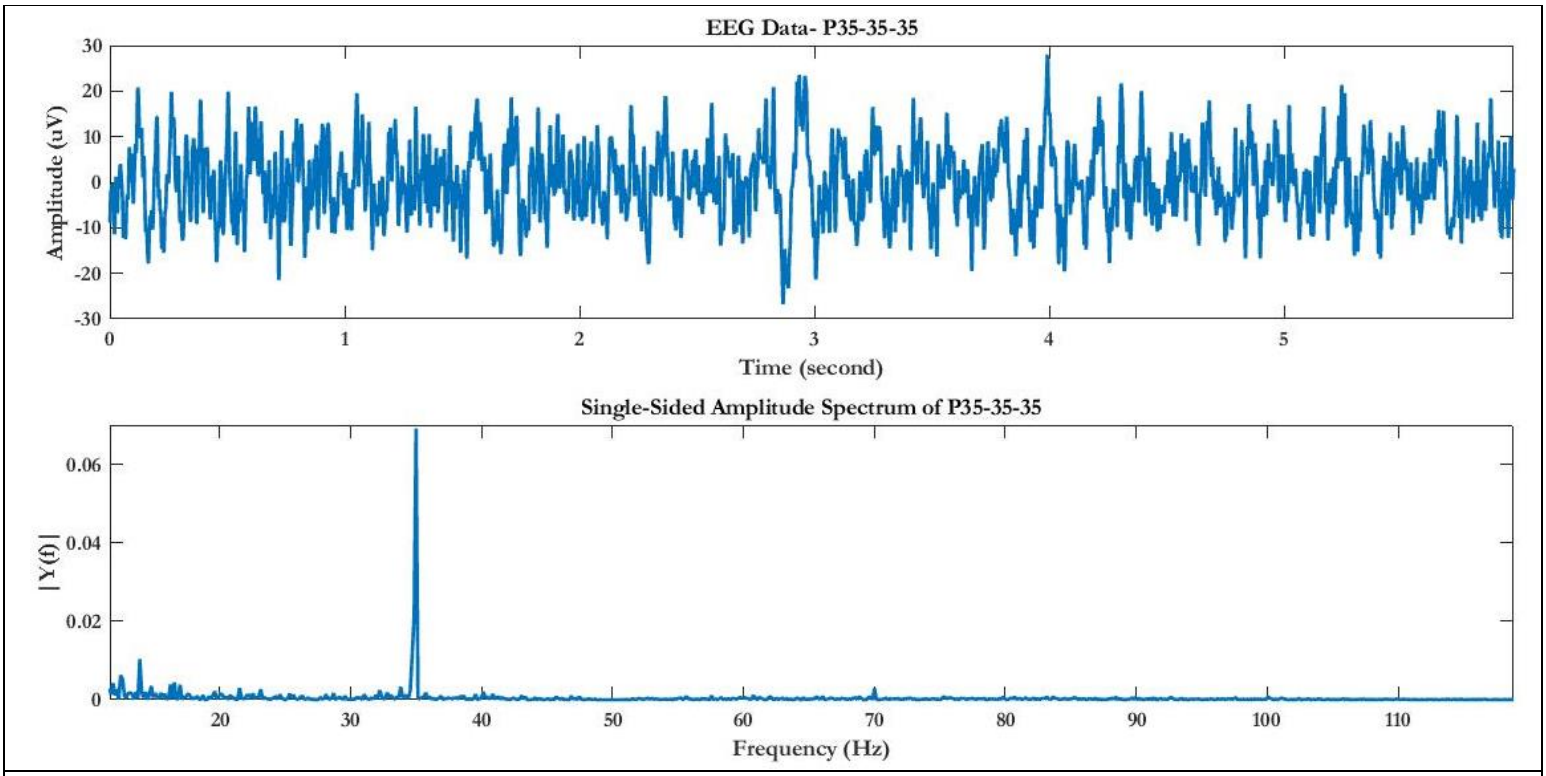
EEG Data- P35-35-35
Amplitude (uV)
Time (second)
Single-Sided Amplitude Spectrum of P35-35-35
|Y(f)|
Frequency (Hz)

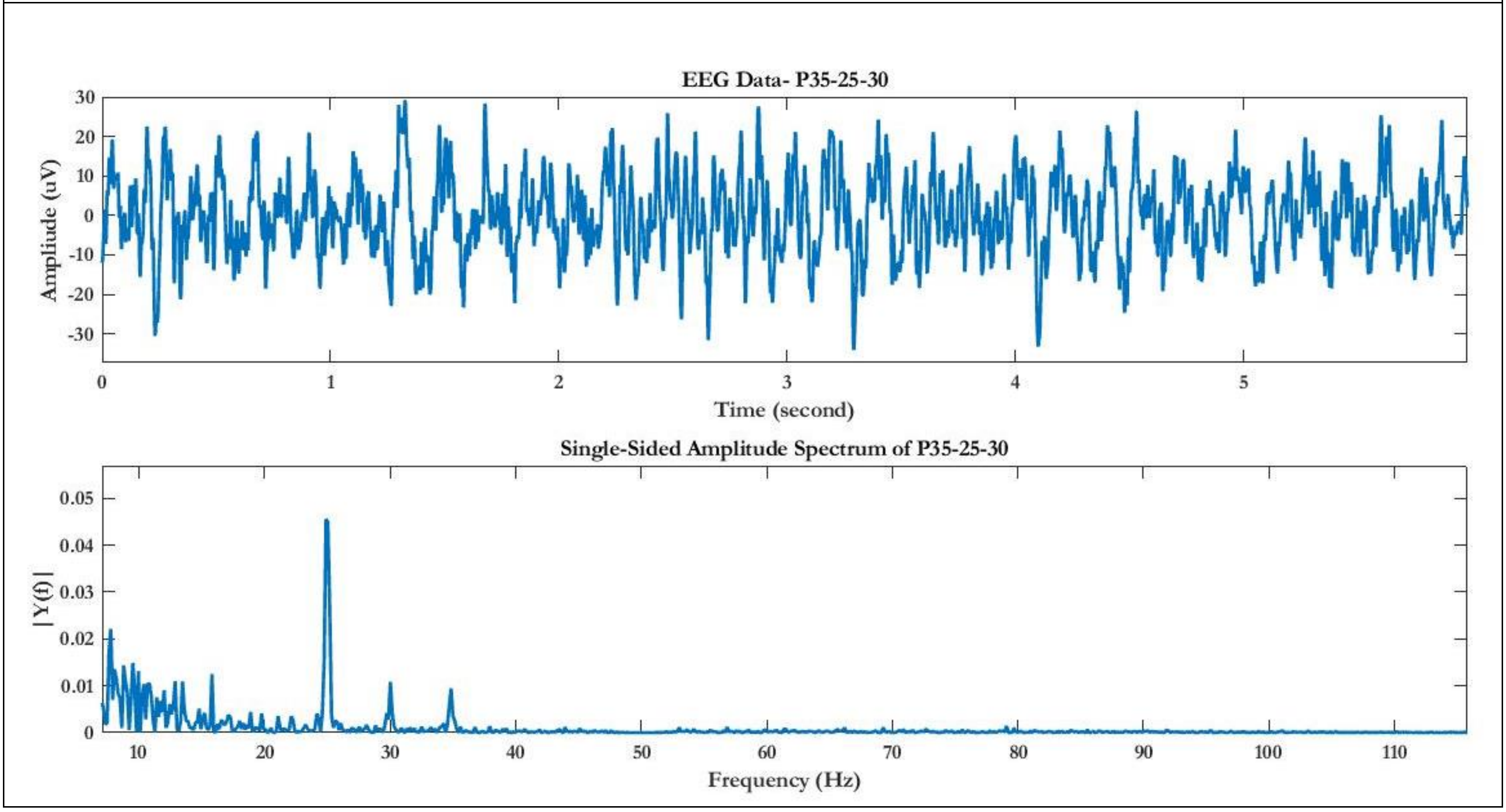
EEG Data- P35-25-30
Amplitude (uV)
Time (second)
Single-Sided Amplitude Spectrum of P35-25-30
|Y(f)|
Frequency (Hz)

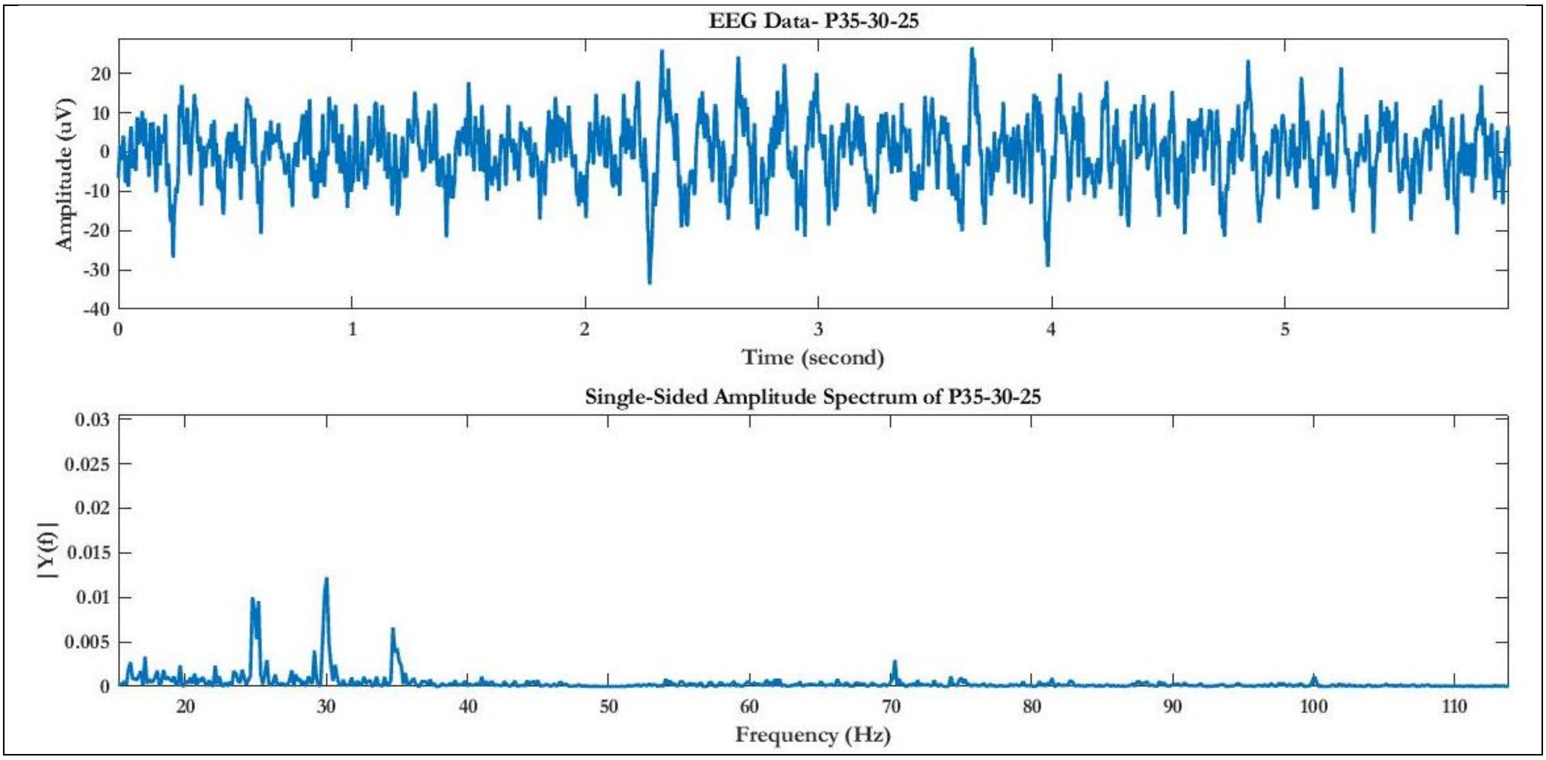
EEG Data- P35-30-25
Amplitude (uV)
Time (second)
Single-Sided Amplitude Spectrum of P35-30-25
|Y(f)|
Frequency (Hz)
